# Genome-wide CRISPR screen identifies RNF24 as a critical host factor for foot-and-mouth disease virus entry

**DOI:** 10.3389/fcell.2026.1909904

**Published:** 2026-08-19

**Authors:** Jinyan Zhang, Hailong Liu, Jian Du, Min Zhang, Mengge Yin, Qiongqiong Zhao, Xinghua Chen, Xiangmin Li, Zengjun Lu, Shengsong Xie, Ping Qian

**Affiliations:** 1 National Key Laboratory of Agricultural Microbiology, Huazhong Agricultural University, Wuhan, Hubei, China; 2 College of Veterinary Medicine, Huazhong Agricultural University, Wuhan, Hubei, China; 3 Key Laboratory of Preventive Veterinary Medicine in Hubei Province, The Cooperative Innovation Center for Sustainable Pig Production, Wuhan, Hubei, China; 4 Key Laboratory of Agricultural Animal Genetics, Breeding and Reproduction, Ministry of Education & Key Lab of Swine Genetics and Breeding, Ministry of Agriculture and Rural Affairs, Huazhong Agricultural University, Wuhan, China; 5 Hubei Jiangxia Laboratory, Wuhan, China; 6 Hubei Hongshan Laboratory, Huazhong Agricultural University, Wuhan, China; 7 State Key Laboratory for Animal Disease Control and Prevention, National Foot-and-Mouth Disease Reference Laboratory, Lanzhou Veterinary Research Institute, Chinese Academy of Agricultural Sciences, Lanzhou, Gansu, China

**Keywords:** CRISPR screening, foot-and-mouth disease virus, LPXN, non-degradative ubiquitination, RNF24

## Abstract

**Background:**

Foot-and-mouth disease virus (FMDV) causes substantial economic losses in global livestock production; however, the key host factors supporting its early infection process remain poorly characterized.

**Methods:**

In this study, we performed an unbiased genome-wide CRISPR/Cas9 knockout screening using porcine cells to screen and identify host factors involved in FMDV infection.

**Results:**

We identified that the E3 ubiquitin ligase RNF24 supports efficient FMDV entry. RNF24 depletion inhibits viral entry and replication, whereas its overexpression enhances viral infectivity. Mechanistically, RNF24 preferentially promotes K27-linked non-degradative polyubiquitination of leupaxin (LPXN) at lysine 162, driving LPXN’s trafficking to the plasma membrane. At the membrane, LPXN assembles a ternary integrin-LPXN-VP1 complex that strengthens virus-receptor interactions and promotes viral adsorption and entry. Disruption of this ubiquitination event via the LPXN K162R mutation impairs complex formation and compromises viral entry.

**Conclusion:**

Together, our study reveals a ubiquitin-dependent RNF24-LPXN regulatory axis that supports FMDV entry, highlights the role of non-degradative ubiquitination in viral pathogenesis, and proposes this interface as a potential target for antiviral intervention.

## Introduction

Foot-and-mouth disease virus (FMDV), an *aphthovirus* genus in the family Picornaviridae (Grubman and Baxt, 2004), is a highly contagious pathogen that infects cloven-hoofed animals, including cattle, swine, and sheep (Jamal and Belsham, 2013). FMDV infection typically manifests as vesicular lesions on the mouth, hooves, and mammary glands, resulting in severe morbidity, reduced productivity, and elevated mortality in young animals (Cabezas et al., 2018; Kitching, 2002; Stenfeldt et al., 2025). Annual economic losses in the global livestock industry due to FMDV are estimated to amount to billions of USD, posing a persistent threat to food security and international trade in animals (Poonsuk et al., 2018). Consequently, a deeper understanding of host-virus interaction mechanisms is crucial for developing effective strategies to control this disease.

The life cycle of FMDV involves a series of coordinated stages: viral entry, genome replication, protein synthesis, progeny virion assembly, and release from host cells (Pulido and Sáiz, 2017; Seago et al., 2013). Viral entry is a critical rate-limiting step that determines viral pathogenicity and tissue tropism (Mercer et al., 2010), making it a focal point for studying viral pathogenesis and designing antiviral strategies (Dejarnac et al., 2018; Klöhn et al., 2024; Wallace et al., 2023). This process is initiated mainly by the binding of the FMDV capsid protein VP1 to integrins and heparan sulfate (Jackson et al., 1996; Wang et al., 2015). Integrins, including αvβ1, αvβ3, αvβ6, and αvβ8, are well-established as key receptors that mediate viral adsorption and internalization (Jackson et al., 2004; Jackson et al., 2002; Jackson et al., 2000; Neff et al., 1998). Their interaction is enabled by the conserved arginine-glycine-aspartic acid (RGD) motif located within the VP1 G-H loop, which serves as the core binding site for integrin subunits (Burman et al., 2006; Fox et al., 1989). Although the structural basis of the direct integrin-VP1 interaction has been well characterized, the host regulatory factors that orchestrate virus-receptor complex assembly at the plasma membrane and modulate the efficiency of viral internalization remain poorly understood. This limited knowledge constitutes a significant barrier to the comprehensive understanding of FMDV pathogenesis.

Viruses are obligate intracellular parasites that rely completely on host cellular machinery and signaling pathways to complete their life cycle (Li et al., 2021; Peng et al., 2020). Identifying host factors essential for viral replication, known as dependency factors, is crucial for deciphering virus-host interaction networks and developing novel antiviral strategies. In the case of FMDV, studies have identified host factors that either promote or restrict viral replication (Liu et al., 2024; Lv et al., 2024; Ma et al., 2025; Yang et al., 2023; Yin M. G. et al., 2024; Zhang et al., 2023). For example, heat shock protein 60 (HSP60) stabilizes the viral non-structural proteins 3 A and 2C, thereby affecting the formation and function of the viral replication complex (Tang et al., 2022). Conversely, ring finger protein 5 (RNF5) interacts with the structural protein VP1 and induces its degradation via ubiquitination at lysine 200, ultimately suppressing viral replication (Zhang et al., 2025).

To comprehensively explore host factors, genome-wide CRISPR/Cas9 screens have emerged as powerful and unbiased tools for the systematic identification of such host factors (Hu et al., 2024; Strich and Chertow, 2019). These functional genetic screens were initially widely applied to viruses within the Flaviviridae family (Marceau et al., 2016; Zhang et al., 2016), such as the Japanese encephalitis virus (JEV), dengue virus (DENV), hepatitis C virus (HCV), and Zika virus (ZIKV), and have identified key regulatory factors, including CALR (Zhao et al., 2020), signal peptide peptidase (Qiao et al., 2025), tripartite motif-containing protein 26 (TRIM26) (Liang et al., 2021), and transmembrane protein 41 B (TMEM41 B) (Liang et al., 2021). With the continuous optimization of CRISPR-based high-throughput screening platforms, CRISPR screening methods are increasingly being used to study host factors involved in *picornaviruses* infections (Bellucci et al., 2025; Kim et al., 2017), including *parechovirus* (PeV) (Qiao et al., 2024) and *enterovirus* 71 (EV71) (Wang et al., 2023). A recent CRISPR screening study demonstrated that TOB1 (Transducer of ERBB2.1) acts as a key host factor for FMDV infection through both IFN- and EGFR-mediated pathways (Peng et al., 2024). Nevertheless, the intricate network of virus-host interactions regulating FMDV early entry remains poorly characterized.

In this study, a genome-wide CRISPR/Cas9 knockout screen identified RNF24 as a key host factor essential for foot-and-mouth disease virus (FMDV) replication. We uncover a previously unknown pathway in which RNF24 promotes K27-linked polyubiquitination of leupaxin (LPXN) at residue K162. This non-degradative modification promotes LPXN translocation to the plasma membrane, where it scaffolds the assembly of an integrin-LPXN-VP1 ternary complex. This complex stabilizes VP1-integrin interaction and enhances viral adsorption and internalization. Our findings establish RNF24 and its substrate LPXN as critical determinants of FMDV entry and delineate a novel non-degradative ubiquitination mechanism that regulates integrin-mediated viral entry. This work advances the understanding of host-FMDV interactions and highlights potential targets for antiviral development.

## Materials and methods

### Cells and virus

Human embryonic kidney 293 T (HEK293 T), swine kidney 6 (SK6), and baby hamster kidney 21 (BHK-21) cells were maintained in Dulbecco’s modified Eagle’s medium (DMEM; Gibco) supplemented with 10% fetal bovine serum (FBS; Gibco), 100 U/mL penicillin (Genview), and 10 μg/mL streptomycin sulfate (Genview). Porcine kidney epithelial cells (LLC-PK1) were cultured in Minimum Essential Medium (MEM; Gibco) containing 10% FBS, 100 U/mL penicillin, and 10 μg/mL streptomycin sulfate. All cells were incubated at 37° C in a humidified 5% CO_2_ atmosphere.

The FMDV O/HN/CHA/93 strain was provided by the National Foot-and-Mouth Disease Reference Laboratory, Lanzhou Veterinary Research Institute, Chinese Academy of Agricultural Sciences. All virological procedures were performed under biosafety level 3 (BSL-3) conditions at the same institute.

### Plasmids construction

The lentiviral sgRNA expression vector was constructed by digesting the lenti-sgRNA-EGFP plasmid with *Bbs* I restriction enzyme to linearize it. Synthesized sgRNA oligonucleotide pairs were then annealed and ligated into the linearized vector. Coding sequences (CDSs) of RNF24, ITGAV, ITGB1, and LPXN were amplified from LLC-PK1 cDNA using gene-specific primers. Truncated and mutant forms of LPXN were generated by overlap PCR with custom-designed primers. RNF24 was cloned into either pTRIP-3Flag or pCMV-Myc vectors, while ITGAV and ITGB1 were inserted into the pCAGGS-HA vector using T4 DNA ligase (Promega, Cat. #M1801). Wild-type (WT), truncated, and mutant LPXN constructs were cloned into pCDNA3.1-3Flag or pCAGGS-HA vectors for downstream expression analysis.

All ubiquitin-related plasmids, including HA-Ub, HA-Ub mutants (K6, K11, K27, K29, K33, K48, K63), Myc-Ub mutants (K6R, K11R, K27R, K29R, K33R, K48R, K63 R), as well as pCAGGS-HA-VP1, pEBG-GST-VP1, pEBG-GST-VP2, pEBG-GST-VP3, and pEBG-GST-VP4, were constructed and maintained in our laboratory. Truncated VP1 variants derived from full-length (FL) VP1 were cloned into the pEBG-GST vector, and FL VP1 was cloned into the pEGFP-C1 vector. All constructs were confirmed by Sanger sequencing (Tsingke). Corresponding primer sequences are listed in Supplementary Table S3.

### Genome-wide CRISPR knockout library construction and FMDV screening

The PigGeCKO v2.0 library was generated as previously described (Liu et al., 2025) by transducing 1 × 10^8^ LLC-PK1-Cas9 cells with sgRNA-library lentiviruses at an MOI of 0.3 in the presence of 10 μg/mL polybrene (Sigma-Aldrich, Cat. # TR-1003). After transduction, EGFP-positive cells were isolated by fluorescence-activated cell sorting (FACS) to remove untransduced cells, and the sorted population was allowed to recover for 7 days. For CRISPR knockout screening, approximately 1 × 10^8^ mutant cells were infected with FMDV at different multiplicities of infection (MOI: 0.00001, 0.001, and 0.01) in serum-free DMEM and incubated at 37° C under 5% CO_2_. After 1.5 h, the inoculum was removed and replaced with fresh DMEM supplemented with 2% FBS and 1% penicillin-streptomycin. Viable cells were harvested at 48 h post-infection, expanded for subsequent rounds of infection, and prepared for deep-sequencing analysis.

### Generation of candidate gene knockout cell lines

SgRNAs targeting candidate genes were cloned into the linearized lenti-sgRNA-EGFP vector. Lentiviruses carrying these sgRNAs were produced following established methods (Kabadi et al., 2014). The resulting sgRNA-expressing lentiviruses were used to transduce LLC-PK1-Cas9 cells. Three days post-transduction, EGFP-positive cells were sorted and collected by FACS. Knockout cells were identified through a sequential procedure: monoclonal cells were lysed, genomic DNA was extracted, the target locus was amplified using gene-specific primers, and the PCR products were verified by Sanger sequencing. Primer sequences used in this assay are listed in Supplementary Table S4.

#### shRNA-mediated gene knockdown assay

To generate LPXN-knockdown cells, three distinct shRNA sequences targeting LPXN were designed and cloned into the pLKO.1 lentiviral vector. Lentiviral particles were produced by co-transfecting HEK293 T cells with the shRNA constructs along with the packaging plasmids psPAX2 and pMD2. G for 48 h. The resulting lentivirus was harvested and used to transduce SK6 cells. The specific shRNA sequences are listed in Supplementary Table S5.

### Plasmid transfection and virus infection

HEK293T, LLC-PK1-Cas9, and SK6 cells were transfected with JetPRIME reagent (Polyplus, USA) according to the manufacturer’s instructions. For viral infection, LLC-PK1 or SK6 cells were seeded in culture plates and grown to approximately 80% confluence. Cells were then inoculated with FMDV at 37° C for 2 h. After viral adsorption, the inoculum was removed, cells were washed with PBS, and fresh medium containing 2% FBS was added, followed by incubation at 37° C under 5% CO_2_ for the indicated durations.

## Reagents and antibodies

Cycloheximide (Cat. # HY-12320) was purchased from MCE (NY, USA). The primary antibodies used in this study included: mouse anti-α-tubulin monoclonal antibody (Cat. # 66031-1-Ig), mouse anti-GAPDH monoclonal antibody (Cat. # 60004-1-Ig), rabbit anti-ATP1A1 polyclonal antibody (Cat. # 14418-1-AP), rabbit anti-FLAG polyclonal antibody (Cat. # 20543-1-AP), rabbit anti-HA polyclonal antibody (Cat. # 51064-2-AP), mouse anti-HA monoclonal antibody (Cat. # 66006-2-Ig), rabbit anti-Myc polyclonal antibody (Cat. # 16286-1-AP), mouse anti-Myc monoclonal antibody (Cat. # 60003-2-Ig), rabbit anti-GST polyclonal antibody (Cat. # 10000-0-AP), mouse anti-GST monoclonal antibody (Cat. # 66001-2-Ig), Rabbit anti-ITGAV monoclonal antibody (Cat. # 84883-5-RR), rabbit anti-ITGB1 polyclonal antibody (Cat. # 12594-1-AP), and rabbit anti-LPXN polyclonal antibody (Cat. # 11307-1-AP) -all from Proteintech; mouse anti-FLAG monoclonal antibody (MBL, Cat. #M185-6); mouse anti-RNF24 monoclonal antibody (Thermo Fisher Scientific, Cat. # PA5-49069); mouse anti-dsRNA monoclonal antibody (Scicons, Cat. # 10010200); and a rabbit anti-FMDV VP1 polyclonal antibody produced and stored in our laboratory.

HRP-conjugated goat anti-mouse IgG (H + L) (Cat. # 330) and HRP-conjugated goat anti-rabbit IgG (H + L) (Cat. # 458) were obtained from MBL (Japan). The following fluorescent secondary antibodies were purchased from Invitrogen: Alexa Fluor Plus 647 goat anti-rabbit IgG (H + L), highly cross-adsorbed (Cat. # A32733); Alexa Fluor 488 goat anti-mouse IgG (H + L), cross-adsorbed (Cat. # A32731); and Alexa Fluor 568 goat anti-rabbit IgG (H + L), cross-adsorbed (Cat. # A11011).

### Western blotting assay

Cells were collected and lysed in NP-40 lysis buffer containing a protease inhibitor cocktail (Beyotime). After incubation on ice for 30 min with intermittent vortexing (three times), lysates were centrifuged at 12,000 × g for 10 min at 4° C to remove debris. The supernatants were denatured by heating in SDS-PAGE loading buffer at 95° C for 10 min and then chilled on ice for 5 min. For immunoblotting, proteins were separated by SDS-PAGE and transferred to polyvinylidene fluoride (PVDF) membranes (Roche, UK). Membranes were blocked with 5% non-fat milk for 2 h at room temperature and incubated with primary antibodies overnight at 4° C, followed by horseradish peroxidase (HRP)-conjugated secondary antibodies for 1 h at room temperature. Protein bands were detected using the Bio-Rad ChemiDoc XRS + imaging system and analyzed with Image Lab software (Bio-Rad, US).

### Quantitative PCR (qPCR)

Total RNA was extracted using TRIzol reagent (Invitrogen, US), and cDNA was synthesized with the HiScript III first Strand cDNA Synthesis Kit (Vazyme, China) following the manufacturer’s instructions. Relative mRNA levels were quantified with SYBR Green master mix (Yeasen, China), and gene expression fold changes were calculated by the comparative ΔΔCT (2^−ΔΔCT^) method. Primer sequences used in the assay are listed in Supplementary Table S6.

### Virus binding and entry assay

For virus binding assays, wild-type and RNF24-knockout LLC-PK1 cells or shNC- and shLPXN-transduced SK6 cells were pre-cooled at 4° C for 30 min, then incubated with FMDV (MOI = 10) at 4° C for 1 h to allow viral attachment. Unbound virus was removed by washing five times with cold PBS. For entry assays, cells were shifted to 37° C for 1 h in fresh complete medium to permit internalization. To remove residual surface-bound virus, cells were treated with cold alkaline high-salt solution (1 M NaCl, 50 mM sodium bicarbonate, pH 9.5) and washed three times, followed by three additional PBS washes. Finally, cells were collected and viral RNA levels were quantified by qPCR.

### Plaque assay

BHK-21 cells were seeded in 6-well plates and grown to approximately 80% confluence. Cells were then incubated with serially diluted viral samples for 2 h at 37° C to allow adsorption. After removal of the inoculum and washing with PBS, an overlay medium consisting of 2% low-melting-point agarose in 2×DMEM supplemented with 4% FBS and 2% penicillin-streptomycin was added to each well. Following 72 h of incubation at 37° C, cells were fixed with 10% formaldehyde and stained with 0.2% crystal violet. Viral titers were calculated by counting plaque-forming units (PFU) and expressed as PFU per mL.

### Coimmunoprecipitation (Co-IP)

The supernatant of the cell lysate was incubated with specific primary antibodies overnight at 4° C with gentle rotation. Protein A/G agarose beads (Beyotime Biotechnology, Cat. #P2028) were then added and incubation continued for an additional 6 h at 4° C. The antigen-antibody-bead complexes were collected by centrifugation at 1,000 × g for 5 min at 4° C, washed five times with ice-cold lysis buffer to remove nonspecifically bound proteins, and finally eluted for analysis by Western blotting.

#### Immunofluorescence assay (IFA)

HEK293T, LLC-PK1, or SK6 cells were fixed with 4% paraformaldehyde for 30 min at room temperature and permeabilized with 0.2% Triton X-100 (Beyotime Biotechnology, Cat. #P0096) for 10 min. After three washes with PBS, cells were blocked with 2% bovine serum albumin (BSA; GENVIEW, Cat. # FA016) in PBS for 1 h at room temperature. Primary antibodies were diluted in PBS containing 2% BSA and incubated with cells overnight at 4° C. Following three PBS washes, cells were incubated with Alexa Fluor-conjugated secondary antibodies for 1 h at room temperature in the dark. After four additional PBS washes, nuclei were stained with DAPI for 10 min at 37° C. Fluorescence images were captured using a Nikon super-resolution microscope.

### Cell fractionation

HEK293 T or LLC-PK1 cells were transfected with plasmids expressing HA-LPXN together with either empty vector control or RNF24-Flag for 24 h. Subsequently, membrane and cytoplasmic proteins were extracted using a commercial fractionation kit (Beyotime, Cat. #P0033) according to the manufacturer’s instructions.

### Statistical analysis

Statistical analyses were conducted using GraphPad Prism software (version 8.0.2). Differences between two groups were assessed using a two-tailed Student’s t-test. The significance threshold was set at *p* < 0.05, with the following notation: *p < 0.05, **p < 0.01, ***p < 0.001, and ****p < 0.0001. Non-significant differences were labeled as “ns” (not significant). Unless otherwise indicated, all data are expressed as mean ± standard deviation (SD).

## Results

### A genome-wide CRISPR/Cas9 knockout screen identified RNF24 as an essential host factor for FMDV infection

**FIGURE 1:**
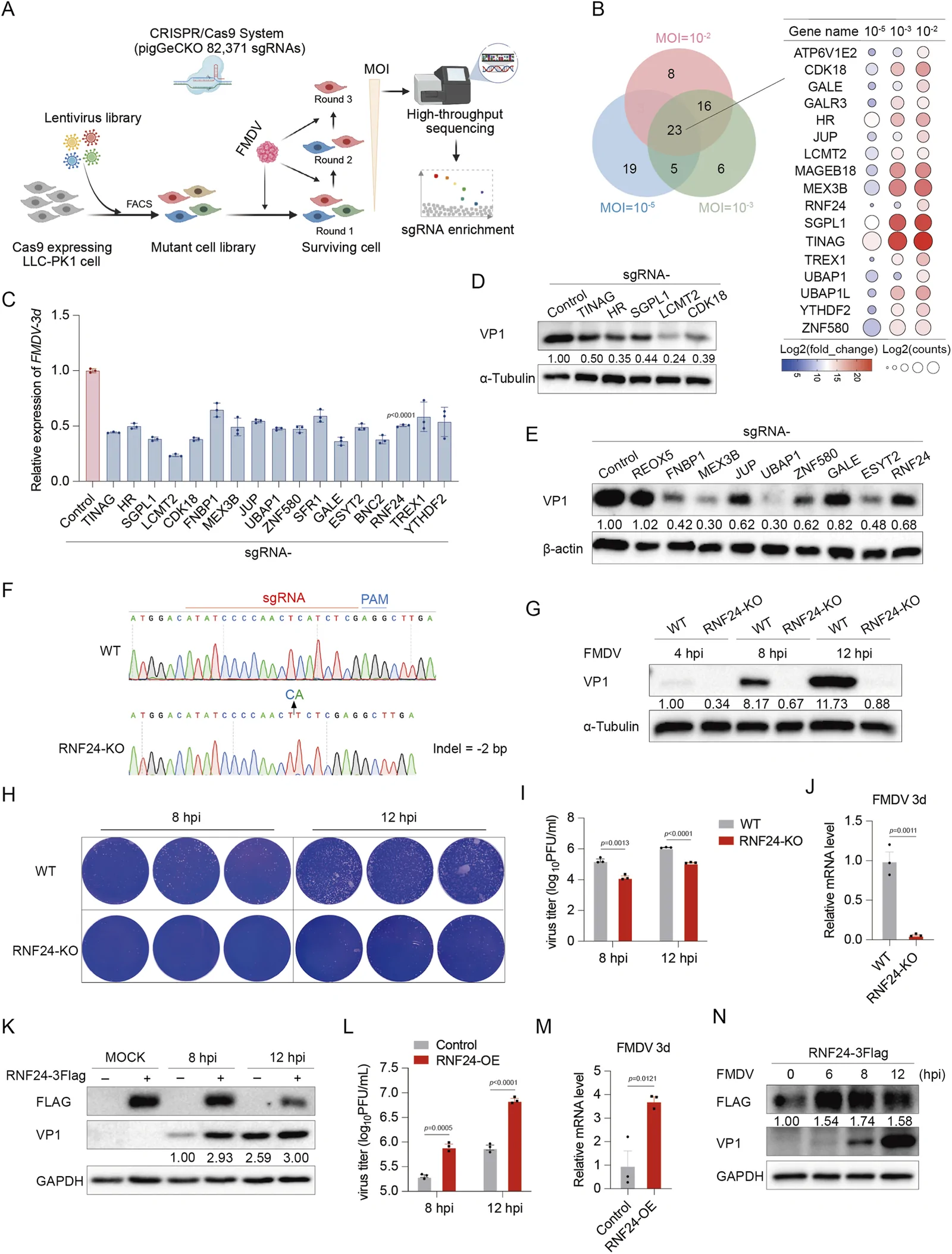
Genome-wide CRISPR screen identifies host factors required for FMDV infection. **(A)** Schematic of the porcine genome-wide CRISPR/Cas9 knockout screen in LLC-PK1-Cas9 cells. Cells were subjected to three rounds of FMDV challenge at increasing MOIs (0.00001, 0.001, 0.01). Surviving cells were collected for sgRNA amplification and sequencing. **(B)** Venn diagram showing the overlap of the top 50 enriched sgRNAs identified across three independent screening experiments. **(C)** Viral RNA levels (qPCR) in pooled mutant cell lines targeting 17 candidate genes following FMDV infection (MOI = 0.01, 12 h), normalized to non-targeting sgRNA controls. **(D,E)** VP1 protein expression (Western blot) in WT and candidate gene-knockout LLC-PK1 cells at 12 h post-infection (MOI = 0.1). Tubulin or actin was used as a loading control. **(F)** Genotyping of homozygous RNF24-KO cells by Sanger sequencing. **(G)** Time-course analysis of VP1 expression (Western blot) in RNF24-KO and WT cells at 4, 8, and 12 h post-infection (MOI = 0.1). **(H,I)** Viral infectivity (plaque assay) in culture supernatants of RNF24-KO and WT cells at 8 h and 12 h post-infection (MOI = 0.1). **(J)** FMDV 3D copy number (qPCR) in RNF24-KO versus WT cells at 12 h post-infection (MOI = 0.1). **(K)** VP1 expression (Western blot) in RNF24-overexpressing (OE) versus control LLC-PK1 cells at 8 h and 12 h post-infection (MOI = 0.1). **(L)** Viral infectivity (plaque assay) in supernatants of RNF24-OE versus control LLC-PK1 cells at 8 h and 12 h post-infection (MOI = 0.1). **(M)** FMDV 3D copy number (qPCR) in RNF24-OE versus control LLC-PK1 cells at 12 h post-infection (MOI = 0.1). **(N)** RNF24-Flag expression (Western blot) in LLC-PK1 cells transfected for 24 h and subsequently infected with FMDV for the indicated times (6, 8, 12 h). The genome-wide screen is specified as two independent biological replicates per selection round (n = 2). All experiments were independently repeated at least three times (n = 3), with representative blots shown. Quantitative data are expressed as mean ± SD. Statistical significance was determined by Student’s t test, and specific p-values are labeled within the figures. Comparisons with p > 0.05 were considered non-significant (ns).

We conducted a genome-wide CRISPR/Cas9 knockout screen in LLC-PK1 cells, a porcine kidney epithelial cell line permissive to FMDV infection, to identify host factors involved in viral entry and replication (Figure 1A). Prior to CRISPR screening, we assessed FMDV-induced cytopathic effects (CPE) in these cells across a range of multiplicities of infection (MOIs: 0, 0.00001, 0.001, and 0.01). Dose-dependent CPE was observed at approximately 24 h post-infection (hpi), with detectable effects even at an MOI as low as 0.00001 (Supplementary Figure S1A). These findings support the high sensitivity of LLC-PK1 cells to FMDV infection and establish their suitability as a model system for this study.

Subsequently, following a previously established methodology (Liu et al., 2025), we generated a genome-wide mutant cell pool by transducing Cas9-expressing LLC-PK1 cells with the PigGeCKO v2 lentiviral knockout library, which contains 82,371 sgRNAs and 1,000 non-targeting controls. The resulting mutant cell library was subsequently challenged with FMDV in three successive rounds of infection, using an MOI of 0.00001 in the first round, 0.001 in the second round, 0.01 in the third round, and each of the three successive selection rounds was performed with two independent biological replicates. Following viral resistant selection, genomic DNA was extracted from surviving mutant cells after three consecutive rounds of infection, and the integrated sgRNAs were amplified by PCR (Figure 1A). The resulting libraries were subjected to deep sequencing on an Illumina NovaSeq 6,000 platform, followed by computational analysis using MAGeCK (Wang et al., 2019; Zajac et al., 2025). The original sgRNA design, plasmid library, sgRNA distribution before and after selection, FMDV deep sequencing data, and MAGeCK statistical outputs (including FDR values) have been compiled in Supplementary Table S1. To identify robust host candidates, the top 50 genes ranked by enrichment score in each screening round were compared. Venn analysis revealed that 23 host genes were consistently enriched across all three rounds (Figure 1B). The recurrence of sgRNAs targeting these genes suggests that they may play key functional roles in FMDV infection.

For functional validation of the overlapping candidate host factors, we constructed pooled mutant cell lines targeting 23 selected genes using CRISPR/Cas9 technology. The mutant cells were infected with FMDV at an MOI of 0.01, and viral replication was evaluated at 12 hpi. Quantitative PCR (qPCR) revealed that the mutation of 17 host factors significantly inhibited FMDV replication (Figure 1C). Consistently, Western blot analysis confirmed a marked reduction in the expression of the FMDV-encoded VP1 protein in these mutant cells (Figures 1D,E). Together, these results support that our genome-wide CRISPR knockout screen conducted in porcine cells effectively identified host factors involved in FMDV infection.

Among the candidate host factors, we selected RNF24 for further investigation due to its function as an E3 ubiquitin ligase with no previously established role in viral infection. Using CRISPR/Cas9 followed by single-cell cloning, we generated an RNF24 homozygous knockout (RNF24-KO) LLC-PK1 cell line. Sanger sequencing confirmed a two-nucleotide deletion in the coding sequence, resulting in a frameshift and loss of RNF24 protein expression (Figure 1F). Next, we compared RNF24-KO and wild-type (WT) cells infected with FMDV at an MOI of 0.1. Viral protein levels were monitored at sequential time points (4, 8, and 12 h) to evaluate the role of RNF24 in FMDV VP1 protein expression. The results indicated that RNF24 knockout significantly suppressed viral protein synthesis (Figure 1G). Viral plaque assays were performed after infection with FMDV at an MOI of 0.1. The results showed that RNF24-KO cells produced fewer and smaller plaques than WT cells at both 8 and 12 hpi (Figures 1H,I). Compared to WT cells, RNF24-KO cells showed a significant reduction in FMDV 3D viral RNA levels, as measured by relative qPCR (Figure 1J).

Having defined the functional impact of RNF24 knockout, we next overexpressed it in WT cells and detected viral replication using Western blotting and plaque assay. Consequently, RNF24-overexpressing cells suggested significantly enhanced FMDV infection, as shown by elevated VP1 protein levels and increased plaque counts at both 8 and 12 hpi (Figures 1K,L). qPCR analysis further supported this finding, revealing a significant increase in the intracellular FMDV genomic copy number in RNF24-overexpressing cells (Figure 1M). Interestingly, RNF24 protein levels were upregulated in LLC-PK1 cells upon FMDV infection (Figure 1N), suggesting that the virus actively induces RNF24 expression to facilitate its replication. Correspondingly, RNF24 knockout inhibited FMDV infection, whereas its overexpression potentiated viral replication, revealing the critical role of RNF24 in promoting FMDV infection.

### RNF24 knockout disrupted FMDV replication during the early stage of its life cycle

**FIGURE 2:**
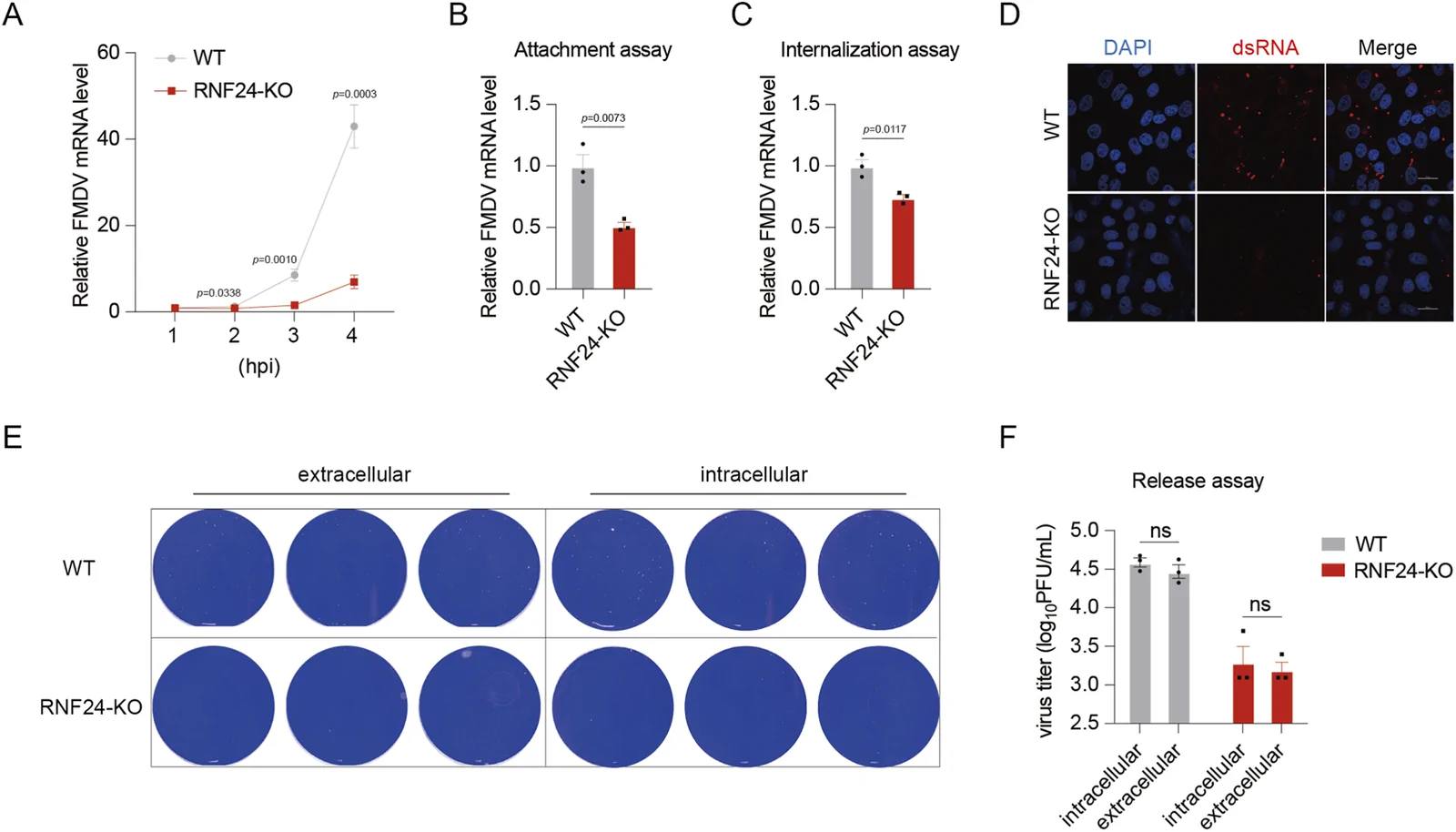
RNF24 knockout impairs FMDV adsorption and internalization. **(A)** FMDV RNA kinetics (qPCR) in RNF24-KO and WT cells from 0 to 4 h post-infection (MOI = 0.1). **(B)** Viral attachment assay. RNF24-KO and WT Cells were incubated with FMDV at 4 °C for 1 h (MOI = 10), washed, and analyzed for bound viral RNA by qPCR. **(C)** Internalization assay. WT and RNF24 KO cells were adsorbed with FMDV (MOI = 10) at 4°C for 1 h. After shifting to 37°C for 1 h to allow entry, cells were subjected to three washes with an ice-cold alkaline high-salt solution (1 M NaCl, 50 mM sodium bicarbonate, pH 9.5) to strip residual surface-bound virions, followed by three final washes with PBS. Internalized viral RNA was measured by qPCR. **(D)** Confocal microscopy detecting dsRNA in RNF24-KO and WT cells at 3 h post-infection (MOI = 10). Scale bars: 20 µm. **(E,F)** Intracellular and extracellular viral titers (plaque assay) in RNF24-KO and WT cells at 12 h post-infection (MOI = 0.1). All experiments were independently repeated at least three times (n = 3), with representative images/blots shown. Quantitative data are expressed as mean ± SD. Statistical significance was determined by Student’s t test, and specific p-values are labeled within the figures. Comparisons with p > 0.05 were considered non-significant (ns).

To identify the specific stage of the FMDV life cycle affected by RNF24 knockout, we first monitored the early phase of viral replication by quantifying FMDV genome copy numbers at hourly intervals during the first round of replication following cellular entry in both RNF24-KO and WT cells. qPCR analysis targeting the FMDV 3 days gene showed a significant decrease in FMDV RNA levels in RNF24-KO cells as early as two hpi, compared with the WT levels (Figure 2A), suggesting that RNF24 deficiency may impair viral entry into host cells. Given that FMDV enters cells rapidly within 1–2 h, and newly synthesized progeny viruses are first detected in the supernatant at 4–6 h post-infection (Grubman and Baxt, 2004), we interpret viral RNA levels at two hpi as primarily reflecting cell-associated or internalized input virus rather than unequivocally representing productive replication. Therefore, the observed reduction in viral RNA at this early time point may suggest a potential impairment in viral entry or the very earliest post-entry events.

Next, we conducted virus binding and internalization assays using a qPCR-based method to quantify the number of FMDV particles attached to the cell surface or internalized into the cytoplasm. The results suggested that RNF24-KO cells exhibited significantly reduced efficiency in both FMDV binding and internalization compared to that in WT cells (Figures 2B,C), suggesting that RNF24 is necessary for efficient viral entry. We further assessed the initial step of viral replication by detecting double-stranded RNA (dsRNA), a key intermediate of FMDV RNA replication, during the early stages of infection. Comparative immunofluorescence analysis (IFA) revealed significantly lower dsRNA levels in RNF24-KO cells at three hpi than in wild-type (WT) cells (Figure 2D), suggesting that RNF24 depletion impairs the initial replication stage of FMDV after viral entry.

To exclude potential effects on viral release, we performed virus release assays. After infecting cells with FMDV, we collected cell supernatants (extracellular virions) and lysed cells (intracellular virions) at specified time points and quantified the viral titers in both fractions using plaque assays. The results indicated no significant differences in viral titers between RNF24-KO and WT cells in either the extracellular or intracellular fractions (Figures 2E,F), demonstrating that RNF24 knockout does not affect the viral release. Taken together, these results indicate that RNF24 plays multifunctional roles in the FMDV life cycle, being required for efficient viral binding, cellular entry, and the subsequent initial RNA replication, but is dispensable for viral release.

### RNF24 facilitates FMDV entry through regulating the integrin-mediated pathway

**FIGURE 3:**
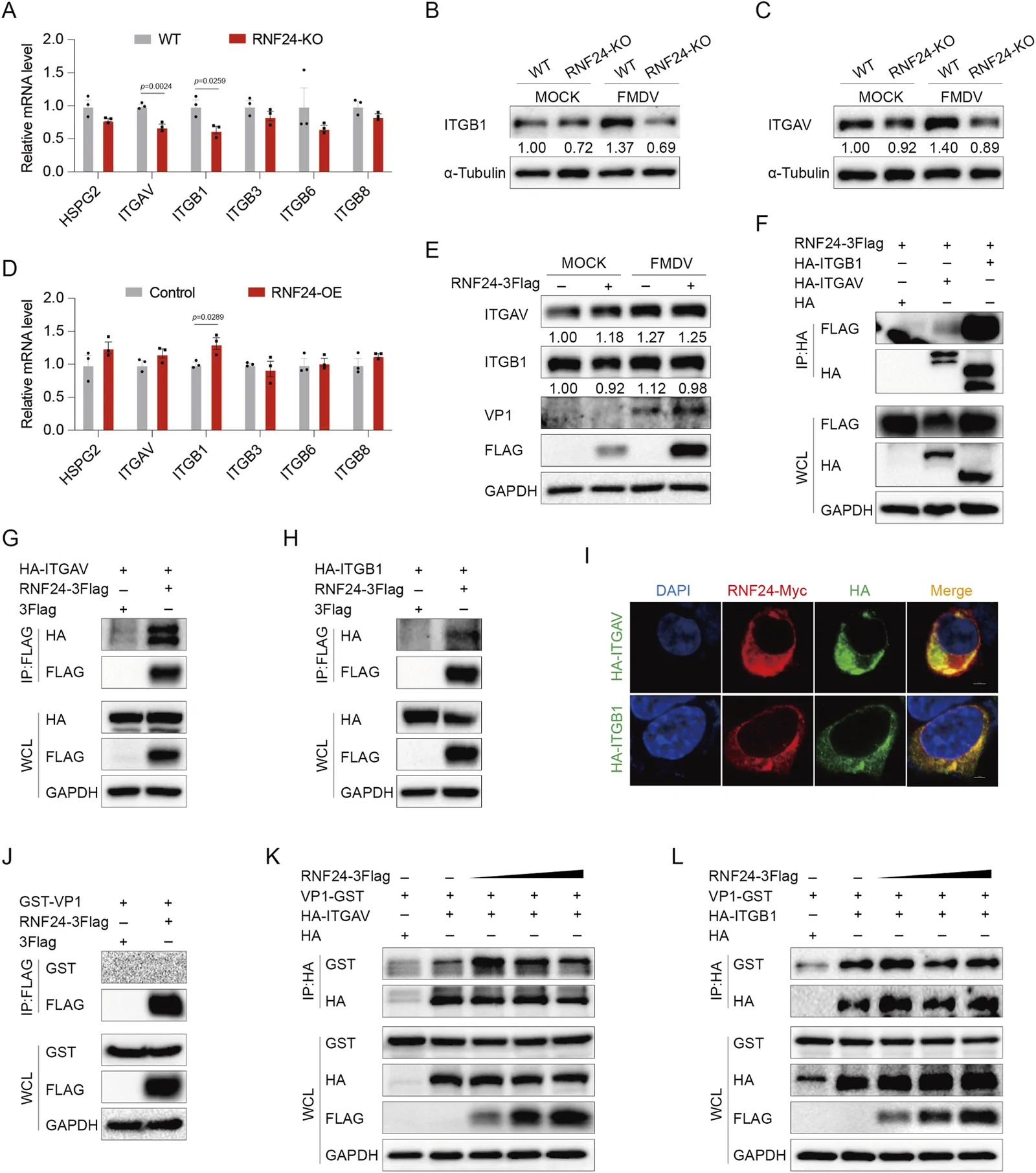
RNF24 modulates integrin-mediated signaling to regulate FMDV entry. **(A)** Transcript levels of HSPGs and integrins in RNF24-KO versus WT cells (qPCR). **(B,C)** Protein levels of ITGB1 **(B)** and ITGAV **(C)** in RNF24-KO and WT cells, with or without FMDV infection (Western blot). **(D)** Transcript levels of HSPGs and integrins in RNF24-overexpressing (OE) versus control LLC-PK1 cells (qPCR). **(E)** Protein levels of ITGAV and ITGB1 in RNF24-OE and control LLC-PK1 cells, with or without FMDV infection (Western blot). **(F-H)** Co-IP analysis in HEK293T cells co-transfected with RNF24-Flag and HA-tagged ITGAV or ITGB1, or corresponding empty vectors for 24 h. **(I)** Confocal microscopy showing colocalization of RNF24-Myc (red) with HA-ITGAV or HA-ITGB1 (green) in HEK293T cells. Scale bars: 2 µm or 5 µm. **(J)** Co-IP analysis in HEK293T cells co-transfected with GST-VP1 and RNF24-Flag or empty vector for 24 h. **(K,L)** Co-IP analysis in HEK293T cells co-transfected with HA-ITGAV **(K)** or HA-ITGB1 **(L)**, GST-VP1, and RNF24-Flag or empty vector for 24 h. All experiments were independently repeated at least three times (n = 3), with representative images/blots shown. Quantitative data are expressed as mean ± SD. Statistical significance was determined by Student’s t test, and specific p-values are labeled within the figures. Comparisons with p > 0.05 were considered non-significant (ns).

Based on the involvement of RNF24 in FMDV entry and its localization to the Golgi membrane (Lussier et al., 2008), we proposed that it might participate in the vesicular transport of membrane receptor proteins and further investigated its influence on FMDV receptors. We observed that knockout of RNF24 significantly reduced the mRNA levels of integrin αv (ITGAV) and integrin β1 (ITGB1), whereas no significant changes were detected in heparan sulfate proteoglycan 2 (HSPG2), ITGB3, ITGB6, or ITGB8 (Figure 3A). Correspondingly, protein levels of ITGAV and ITGB1 were also decreased in RNF24-KO cells compared with WT cells (Figures 3B,C). In contrast, overexpression of RNF24 in LLC-PK1 cells did not significantly alter the mRNA or protein levels of ITGAV and ITGB1 (Figures 3D,E).

Although RNF24 localizes to the Golgi apparatus, we postulated that it interacts with integrin subunits ITGAV and ITGB1 to modulate their functions. To test this, we performed Co-IP assays, which suggested that RNF24 interacts with both ITGAV and ITGB1 (Figures 3F–H). Confocal microscopy further revealed the colocalization of RNF24 with ITGAV and ITGB1 (Figure 3I), supporting a physical interaction between RNF24 and the integrin subunits. However, Co-IP assays showed no detectable interaction between RNF24 and VP1 (Figure 3J), suggesting that RNF24 indirectly regulates FMDV entry without binding to the viral VP1 protein.

Considering that RNF24 overexpression did not alter the expression levels of ITGAV and ITGB1, and given prior evidence suggesting that RNF24 may regulate the membrane incorporation of ion channel proteins (Lussier et al., 2008), we next asked whether RNF24 overexpression influences the interaction between VP1 and integrins. Co-IP assays showed that RNF24 overexpression significantly enhanced VP1 binding to both ITGAV and ITGB1. However, this enhancing effect did not exhibit a clear dose-dependent relationship across the tested range of RNF24 overexpression plasmid concentrations (Figures 3K,L).

Together, these results indicate that RNF24 promotes FMDV cellular entry by interacting with ITGAV and ITGB1 and enhancing their binding to the viral VP1 protein. Furthermore, RNF24 is necessary to maintain the normal expression levels of these integrin subunits, while its overexpression regulates integrin function via a mechanism that is independent of transcriptional control and protein expression.

### RNF24 interacts with LPXN, a regulatory factor in the integrin signaling pathway

**FIGURE 4:**
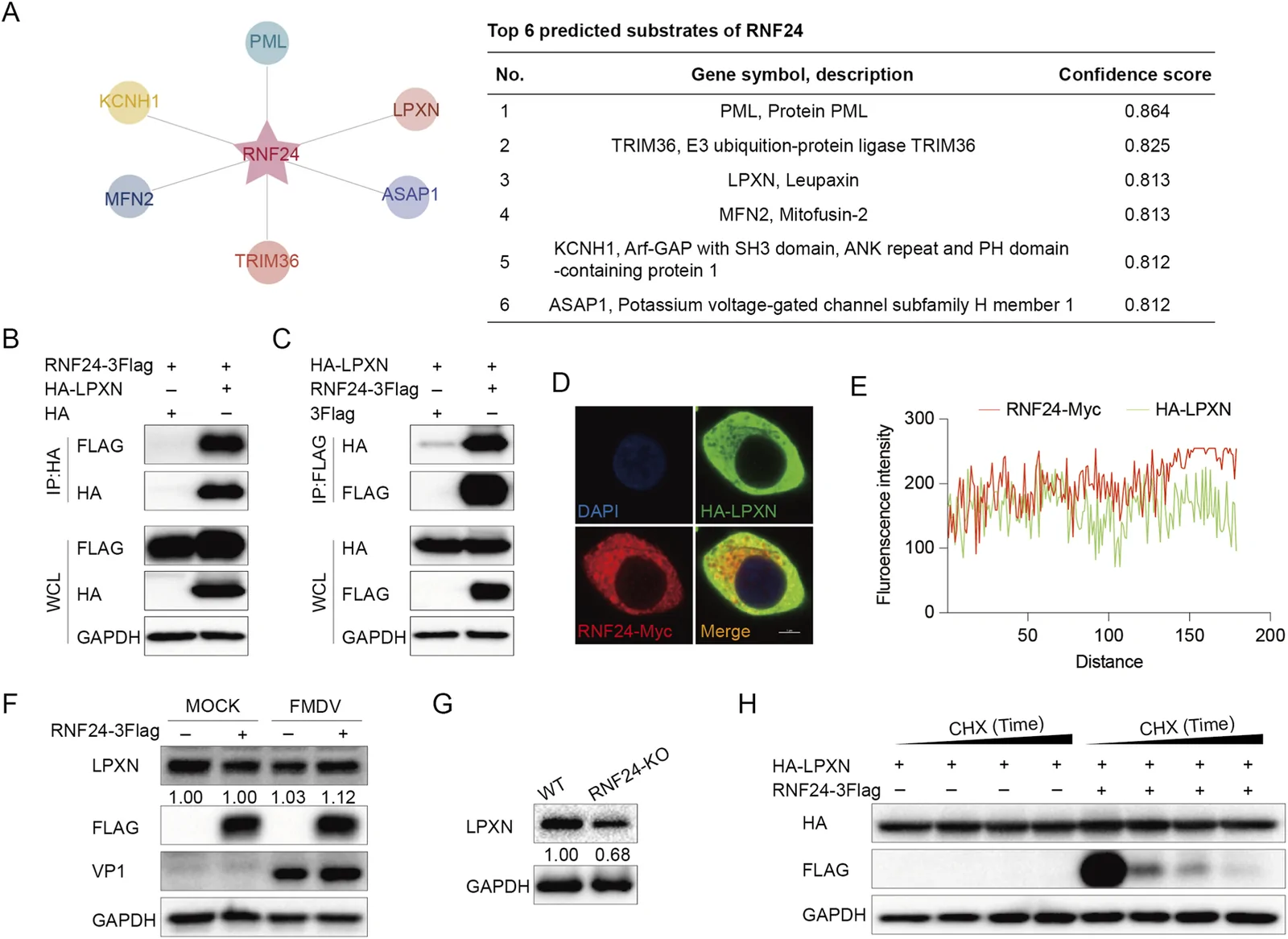
RNF24 interacts with LPXN. **(A)** Potential ubiquitination substrates of RNF24 predicted by UbiBrowser 2.0. **(B,C)** Co-IP analysis in HEK293T cells co-transfected with HA-LPXN and RNF24-Flag or empty vector for 24 h. **(D)** Confocal microscopy showing colocalization of RNF24-Myc (red) with HA-LPXN (green) in HEK293T cells. Scale bar: 5 µm. **(E)** Quantification of fluorescence intensity at RNF24 and LPXN colocalized sites. **(F)** LPXN expression (Western blot) in RNF24-overexpressing LLC-PK1 cells with or without FMDV infection. **(G)** LPXN expression (Western blot) in RNF24-KO and WT cells. **(H)** LPXN protein stability assay. HA-LPXN and RNF24-Flag were co-expressed in HEK293T cells, followed by cycloheximide (CHX) chase treatment for the indicated durations and Western blot analysis. All experiments were independently repeated at least three times (n = 3), with representative images/blots shown.

As RNF24 belongs to the ring finger protein family, which represents one of the largest E3 ubiquitin ligase families (Cai et al., 2022), but its ubiquitination function has not been reported, we further investigated whether RNF24 regulates the integrin pathway via its ubiquitination activity. Using UbiBrowser 2.0 for substrate prediction, we identified LPXN, a negative regulator of integrin-mediated cell adhesion and migration (Klapproth et al., 2019; Tanaka et al., 2010), as a potential ubiquitination substrate of RNF24 (Figure 4A). Since LPXN is a known regulator of the integrin pathway and has been reported to interact with integrin α4 (ITGA4) (Liu et al., 1999), we next examined whether LPXN influences the expression of ITGAV and ITGB1. Our results showed that LPXN overexpression significantly increased the protein levels of both ITGAV and ITGB1 (Supplementary Figures S2A–S2B). Co-IP assays supported that LPXN interacts with both ITGAV and ITGB1 (Supplementary Figure S2C–E), and confocal microscopy further validated their colocalization (Supplementary Figures S2F–S2G). Together, these results support that LPXN is involved in regulating ITGAV and ITGB1.

Based on these findings, we proposed that RNF24 modulates the integrin pathway through LPXN targeting. To test this, we examined whether RNF24 interacts with LPXN using Co-IP assays. Reciprocal Co-IP suggested a physical interaction between RNF24 and LPXN (Figures 4B,C). Confocal microscopy further revealed the colocalization of RNF24 and LPXN in the cytoplasm (Figures 4D,E), supporting a functional interplay between the two proteins. Since LPXN was predicted as a ubiquitination substrate of RNF24 and ubiquitination commonly targets proteins for proteasomal degradation (Dikic, 2017; Wang et al., 2024), we initially proposed that RNF24 enhances integrin signaling by promoting LPXN degradation. To test this, we examined the effect of RNF24 overexpression on LPXN protein levels. Unexpectedly, Western blot analysis showed that RNF24 overexpression did not significantly alter LPXN expression (Figure 4F). In contrast, and consistent with the interaction data, RNF24 knockout markedly reduced LPXN protein levels (Figure 4G).

To further determine whether RNF24 regulates LPXN in a degradation-independent manner, we assessed the effect of RNF24 on LPXN protein stability using cycloheximide (CHX) chase assay. The results showed that RNF24 did not significantly affect LPXN stability, and LPXN exhibited a long half-life in cells (Figure 4H). Together, these findings suggest that RNF24 interacts with LPXN and modulates its expression via a non-degradative mechanism.

### RNF24 promotes K27-linked ubiquitination of LPXN at K162 residue

**FIGURE 5:**
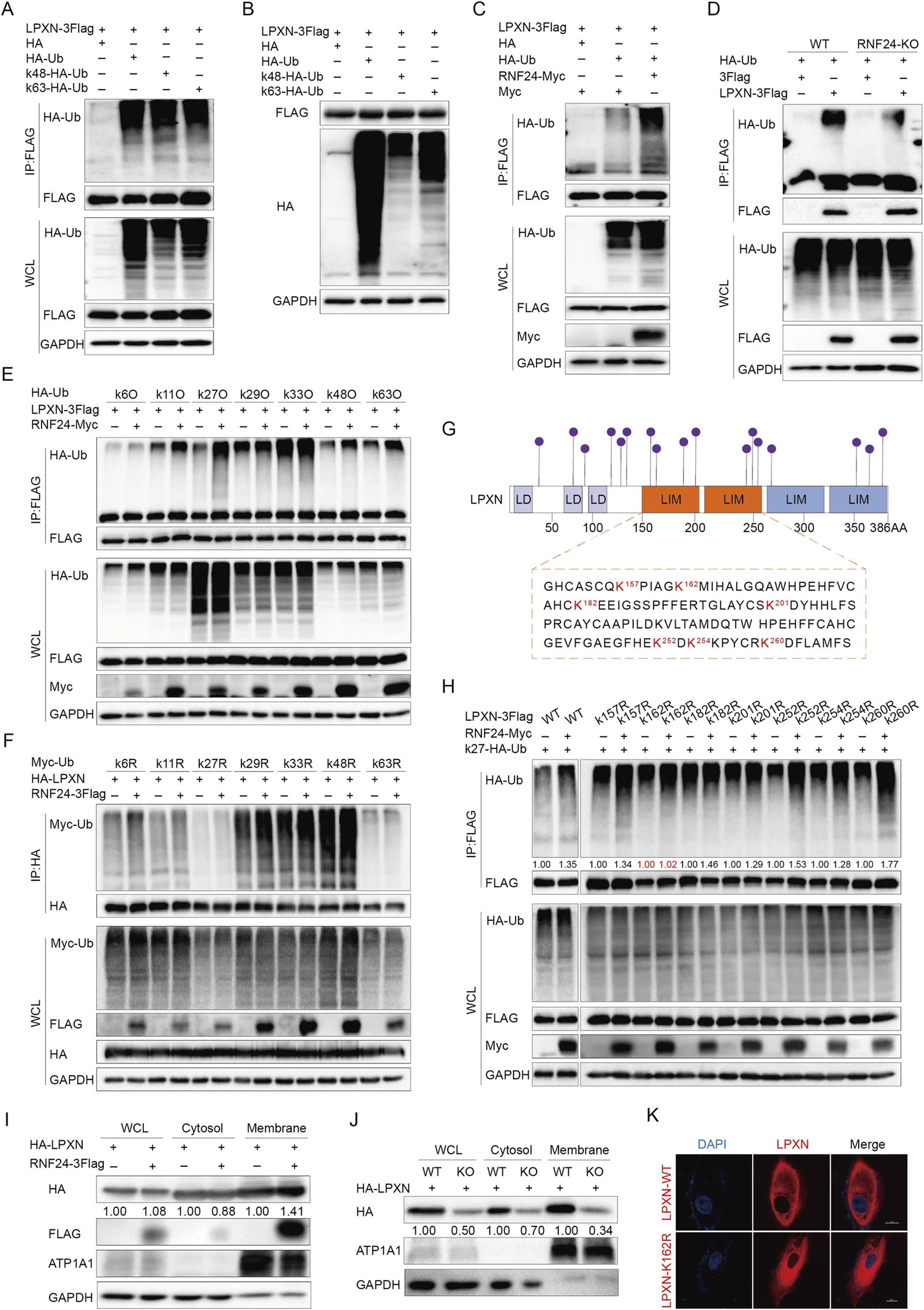
RNF24 preferentially promotes K27-linked ubiquitination of LPXN at residue K162. **(A)** Co-IP analysis in HEK293T cells co-transfected with LPXN-Flag and HA-ubiquitin (HA-Ub; WT or indicated mutants) or empty vector for 24 h. **(B)** Immunoblot analysis of lysates from HEK293T cells expressing LPXN-Flag and HA-Ub (WT or mutants) for 24 h. **(C)** Co-IP analysis in HEK293T cells co-expressing HA-Ub, LPXN-Flag, and Myc-RNF24 or empty vector for 24 h. **(D)** Co-IP analysis in RNF24-KO versus WT cells co-transfected with HA-Ub, Flag-LPXN, and Myc-RNF24 or empty vector for 24 h. **(E)** Co-IP analysis in HEK293T cells expressing HA-Ub (WT or mutants), Flag-LPXN, and Myc-RNF24 or empty vector for 24 h. **(F)** Co-IP analysis in HEK293T cells expressing Myc-Ub (WT or mutants), HA-LPXN, and RNF24-Flag or empty vector for 24 h. **(G)** Schematic diagram of LPXN ubiquitination sites and point mutations. **(H)** Co-IP analysis in HEK293T cells expressing HA-Ub, LPXN-Flag (WT or mutants), and RNF24-Myc or empty vector for 24 h. **(I)** Subcellular fractionation and Western blot analysis of HA-LPXN in cytosolic and membrane fractions from HEK293T cells expressing HA-LPXN with or without RNF24-Flag for 24 h. **(J)** Subcellular fractionation and Western blot analysis of HA-LPXN in cytosolic and membrane fractions from RNF24-KO versus WT cells transfected with HA-LPXN for 24 h. **(K)** Confocal microscopy showing subcellular localization of HA-LPXN (WT or mutants; red) in HEK293T cells. Scale bar: 10 µm. All experiments were independently repeated at least three times (n = 3), with representative images/blots shown.

To clarify how RNF24 regulates LPXN, we examined whether RNF24 influences LPXN ubiquitination, a modification that has not been previously reported for LPXN. First, we confirmed that LPXN undergoes ubiquitination by transfecting HEK293 T cells with plasmids encoding HA-tagged WT ubiquitin (HA-Ub), K48-linked ubiquitin mutant, or K63-linked ubiquitin mutant, together with LPXN-Flag or empty control vector, followed by Co-IP assay for subsequent analysis. (Figure 5A). Surprisingly, subsequent analysis indicated that ubiquitination did not alter LPXN protein levels (Figure 5B). We then assessed the role of RNF24 in LPXN ubiquitination by co-transfecting HEK293 T cells with HA-Ub, LPXN-Flag, and either Myc-RNF24 or empty control vector. RNF24 overexpression strongly increased LPXN ubiquitination (Figure 5C), whereas RNF24 knockout decreased it (Figure 5D), together supporting that RNF24 positively regulates LPXN ubiquitination.

To further determine whether the E3 ubiquitin ligase activity of RNF24 is required for LPXN ubiquitination, we generated ligase-dead mutants by introducing site-directed mutations within its RING domain. Notably, the quadruple mutant (C78A/C81A/H98A/H101 A) failed to promote LPXN ubiquitination (Supplementary Figure S3A), and this mutant was also unable to rescue the reduction in LPXN ubiquitination levels caused by RNF24 knockout (Supplementary Figure S3B), indicating that RNF24 promotes LPXN ubiquitination via its intrinsic E3 ubiquitin ligase activity. Furthermore, in contrast to wild-type RNF24, the RING-domain mutant failed to enhance FMDV replication in LLC-PK1 cells (Supplementary Figure S3C), suggesting that the E3 ligase activity of RNF24 is also critical for its pro-viral function.

To determine the linkage specificity of RNF24-mediated ubiquitination of LPXN, cells were co-transfected with plasmids expressing Myc-RNF24, LPXN-Flag, and individual ubiquitin mutants (K6, K11, K27, K29, K33, K48, and K63). Co-IP results showed that among the ubiquitin mutants tested, RNF24 preferentially promoted K27-linked polyubiquitination of LPXN, with no significant enhancement observed for other linkage types (Figure 5E). To further validate this finding, we used a panel of ubiquitin mutants in which each lysine was individually substituted with arginine (K6R, K11R, K27R, K29R, K33R, K48R, or K63 R). Strikingly, mutation of the K27 residue completely abolished the RNF24-induced increase in LPXN ubiquitination (Figure 5F), suggesting that RNF24 primarily mediates K27-linked polyubiquitination of LPXN.

LPXN contains 24 lysine residues. To identify the specific lysine residues targeted by RNF24 for K27-linked polyubiquitination, we first performed ubiquitinome analysis, which identified 17 potential ubiquitination sites on LPXN, namely, K36, K79, K86, K116, K121, K125, K157, K162, K182, K201, K252, K254, K260, K269, K364, K373, and K382, all of which were identified with high confidence. (Supplementary Table S2). By co-transfecting HA-Ub, LPXN truncation mutants, and RNF24 into HEK293 T cells, we narrowed down the candidate sites and found that RNF24 mediates ubiquitination within the LIM1 and LIM2 domains of LPXN (Figure 5G; Supplementary Figure S4A-C). We then focused on seven candidate lysines within these domains (K157, K162, K182, K201, K252, K254, and K260) and constructed the corresponding LPXN mutants by replacing lysine with arginine. Among them, the mutation of lysine 162 to arginine (K162R) significantly reduced RNF24-mediated ubiquitination (Figure 5H), indicating that RNF24 promotes K27-linked polyubiquitination of LPXN specifically at K162 site.

Considering the diverse functions of K27-linked ubiquitination and the documented localization of LPXN to both the plasma membrane and cytoplasm (Gupta et al., 2003; Sundberg-Smith et al., 2008), we proposed that RNF24 modulates the subcellular distribution of LPXN via K27-linked ubiquitination. Membrane fractionation assays showed that RNF24 overexpression increased LPXN localization at the plasma membrane (Figure 5I). Conversely, RNF24 knockout decreased LPXN levels in both the cytoplasmic and membrane fractions (Figure 5J). Confocal microscopy analysis of ectopically expressed K162R-mutant versus WT LPXN in HEK293 T cells provided consistent evidence that WT LPXN displayed clear membrane enrichment, whereas the K162R mutant showed diffuse cytoplasmic distribution (Figure 5K).

Taken together, these findings indicate that RNF24 interacts with LPXN and promotes its K27-linked polyubiquitination at K162 within the LIM1/LIM2 domains. LPXN RNF24-mediated ubiquitination facilitates LPXN intracellular translocation and enhances its localization to the plasma membrane.

### LPXN, a ubiquitination substrate of RNF24, plays a critical role in FMDV entry

**FIGURE 6:**
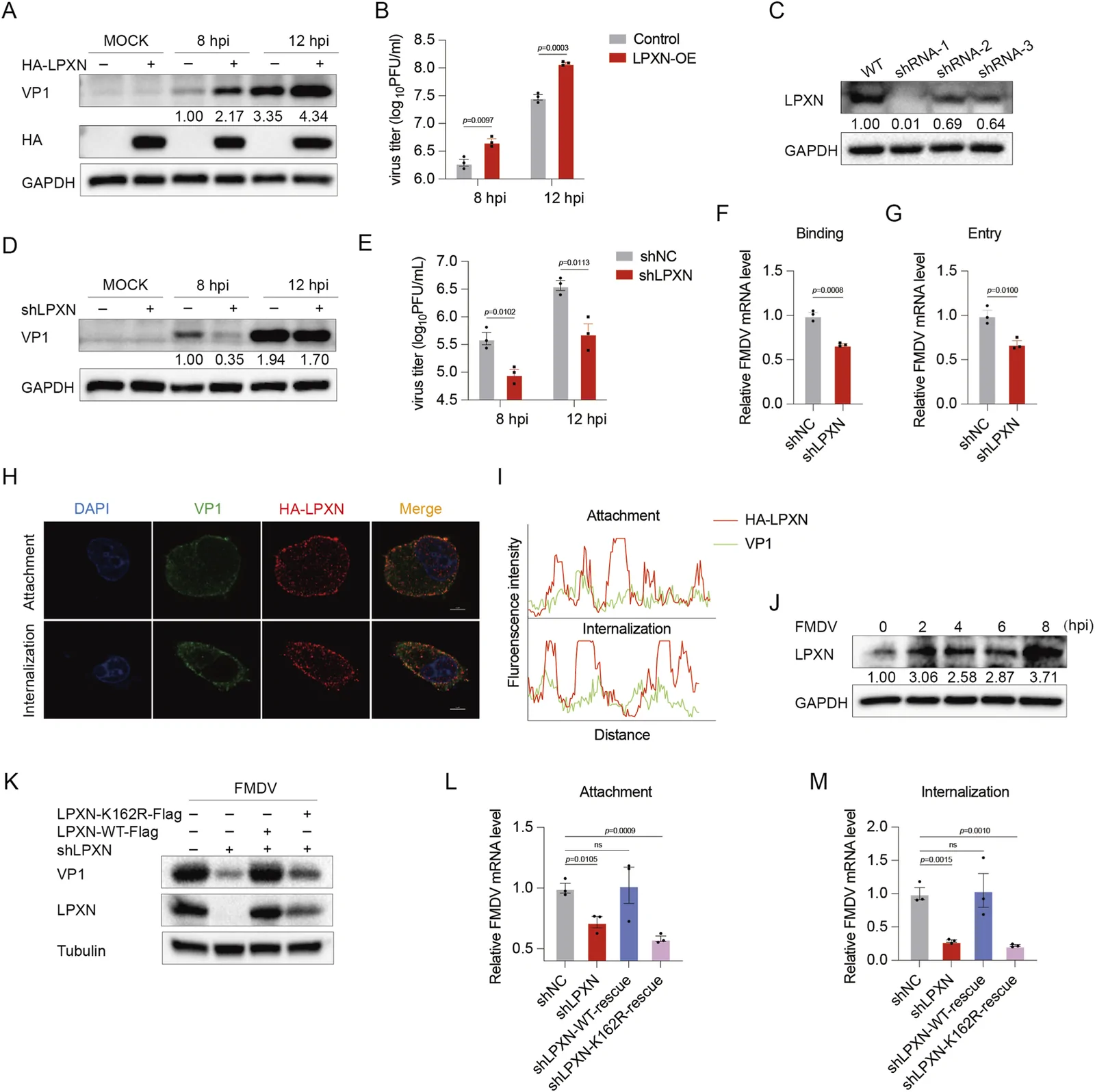
LPXN is required for FMDV entry. **(A)** VP1 expression (Western blot) in LPXN-overexpressing (OE) and control SK6 cells at 12 h post-infection (MOI = 0.1). **(B)** Viral infectivity (plaque assay) in supernatants of LPXN-OE and control SK6 cells at 12 h post-infection (MOI = 0.1). **(C)** LPXN protein levels (Western blot) in SK6 cells at 48 h after transduction with shRNA lentiviruses targeting LPXN. **(D)** VP1 expression (Western blot) in shLPXN and shNC transduced SK6 cells at 12 h post-infection (MOI = 0.1). **(E)** Viral infectivity (plaque assay) in supernatants of shLPXN and shNC transduced SK6 cells at 12 h post-infection (MOI = 0.1). **(F)** Viral attachment assay. shLPXN and shNC transduced SK6 cells were incubated with FMDV (MOI = 10) at 4 °C for 1 h, washed, and analyzed for bound viral RNA by qPCR. **(G)** Internalization assay. shLPXN and shNC transduced SK6 cells were adsorbed with FMDV (4 °C, 1 h, MOI = 10), After shifting to 37°C for 1 h to allow entry, cells were subjected to three washes with an ice-cold alkaline high-salt solution (1 M NaCl, 50 mM sodium bicarbonate, pH 9.5) to strip residual surface-bound virions, followed by three final washes with PBS. Internalized viral RNA was measured by qPCR. **(H)** Confocal microscopy showing colocalization of HA-LPXN (red) with VP1 (green) in FMDV-infected SK6 cells (MOI = 10). Cells were processed as in panels F and G before staining and imaging. **(I)** Fluorescence intensity quantification at sites of LPXN-VP1 colocalization. **(J)** Immunoblot analysis of SK6 cell lysates after 12 h of FMDV infection (MOI = 0.1) using the indicated antibodies. **(K)** VP1 expression detected by Western blot in SK6 cells transduced with shNC or shLPXN, with shLPXN cells further complemented by re-expression of wild-type LPXN (WT) or the K162R mutant, at 12 h post-infection with FMDV (MOI = 0.1). **(L)** Viral attachment assay in SK6 cells transduced with shNC or shLPXN, with shLPXN cells reconstituted with wild-type LPXN (WT) or the K162R mutant. Cells were incubated with FMDV at 4°C for 1 h to allow binding, and attached viral RNA was quantified by RT-qPCR. Data are presented as relative attachment efficiency normalized to the shNC group. **(M)** Viral internalization assay in SK6 cells transduced with shNC or shLPXN, with shLPXN cells reconstituted with wild-type LPXN (WT) or the K162R mutant. Cells were incubated with FMDV at 4°C for 1 h to allow binding, shifted to 37°C for 1 h to permit internalization, and surface-bound viruses were removed by ice-cold alkaline high-salt solution wash. Internalized viral RNA was quantified by RT-qPCR. Data are presented as relative entry efficiency normalized to the shNC group. All experiments were independently repeated at least three times (n = 3), with representative images/blots shown. Quantitative data are expressed as mean ± SD. Statistical significance was determined by Student’s t test, and specific p-values are labeled within the figures. Comparisons with p > 0.05 were considered non-significant (ns).

As LPXN acts as a downstream target of RNF24 and regulates integrin signaling, we next examined whether LPXN plays a direct and essential role in FMDV replication, a function not previously linked to this protein. Swine kidney 6 (SK6) cells were first transiently transfected with Flag-LPXN overexpression plasmid or empty vector control, followed by infection with FMDV at an MOI of 0.1 for 24 h. Overexpression of LPXN significantly elevated VP1 protein levels at multiple time points relative to vector control (Figure 6A). Plaque assays suggested that LPXN overexpression significantly increased the FMDV titers (Figure 6B), indicating that LPXN promotes viral replication.

Using an RNA interference assay, we found that endogenous LPXN protein levels were effectively reduced by shLPXN-1 (Figure 6C). The cells were then infected with FMDV at an MOI of 0.1. LPXN knockdown markedly decreased VP1 protein levels at 8 and 12 hpi compared to shNC-transfected controls (Figure 6D). Plaque assays further suggested that LPXN knockdown significantly reduced viral titers relative to the shNC-transfected controls (Figure 6E). Furthermore, we performed qPCR-based attachment and internalization assays to quantify cell-surface-bound and internalized FMDV particles separately. LPXN knockdown significantly reduced both FMDV attachment and internalization compared to shNC-transfected controls (Figures 6F,G), directly demonstrating that LPXN is necessary for efficient viral entry.

To further examine the role of LPXN in FMDV entry, we analyzed the colocalization of LPXN and FMDV VP1 during viral adsorption and internalization using confocal microscopy. SK6 cells transfected with shLPXN or shNC were infected with FMDV at an MOI of 10, and immunofluorescence staining was performed at one hpi (adsorption phase) and two hpi (internalization phase). Confocal microscopy revealed the colocalization of LPXN with FMDV VP1 at both time points (Figures 6H,I). This dynamic colocalization supports the involvement of LPXN in FMDV entry.

We also examined whether LPXN expression was altered following FMDV infection. Infection with FMDV at an MOI of 0.1 significantly upregulated endogenous LPXN protein levels at multiple time points compared to mock-infected- cells (Figure 6J), suggesting that FMDV may induce LPXN expression to facilitate viral replication. Taken together, these findings support that LPXN is an essential host factor for efficient FMDV replication, specifically promoting viral entry into the host cells.

To further evaluate the functional significance of RNF24-mediated ubiquitination at the K162 residue of LPXN in FMDV replication and entry, we performed rescue experiments in shLPXN-treated SK6 cells by re-expressing WT LPXN or the K162R mutant, followed by detection of FMDV VP1 expression. Notably, in contrast to WT LPXN, the K162R mutant failed to effectively reverse the inhibitory effect of LPXN knockdown on FMDV infection (Figure 6K), indicating that the K162 site is critical for LPXN-mediated promotion of viral infection. Subsequent attachment and internalization assays further revealed that the K162R mutant was unable to rescue the defects in FMDV attachment and entry caused by LPXN depletion (Figures 6L,M), demonstrating that the K162 residue of LPXN is also indispensable for its role in FMDV entry.

### RNF24 promotes the assembly of the integrin-lpxn-vp1 ternary complex at the plasma membrane during FMDV infection

**FIGURE 7:**
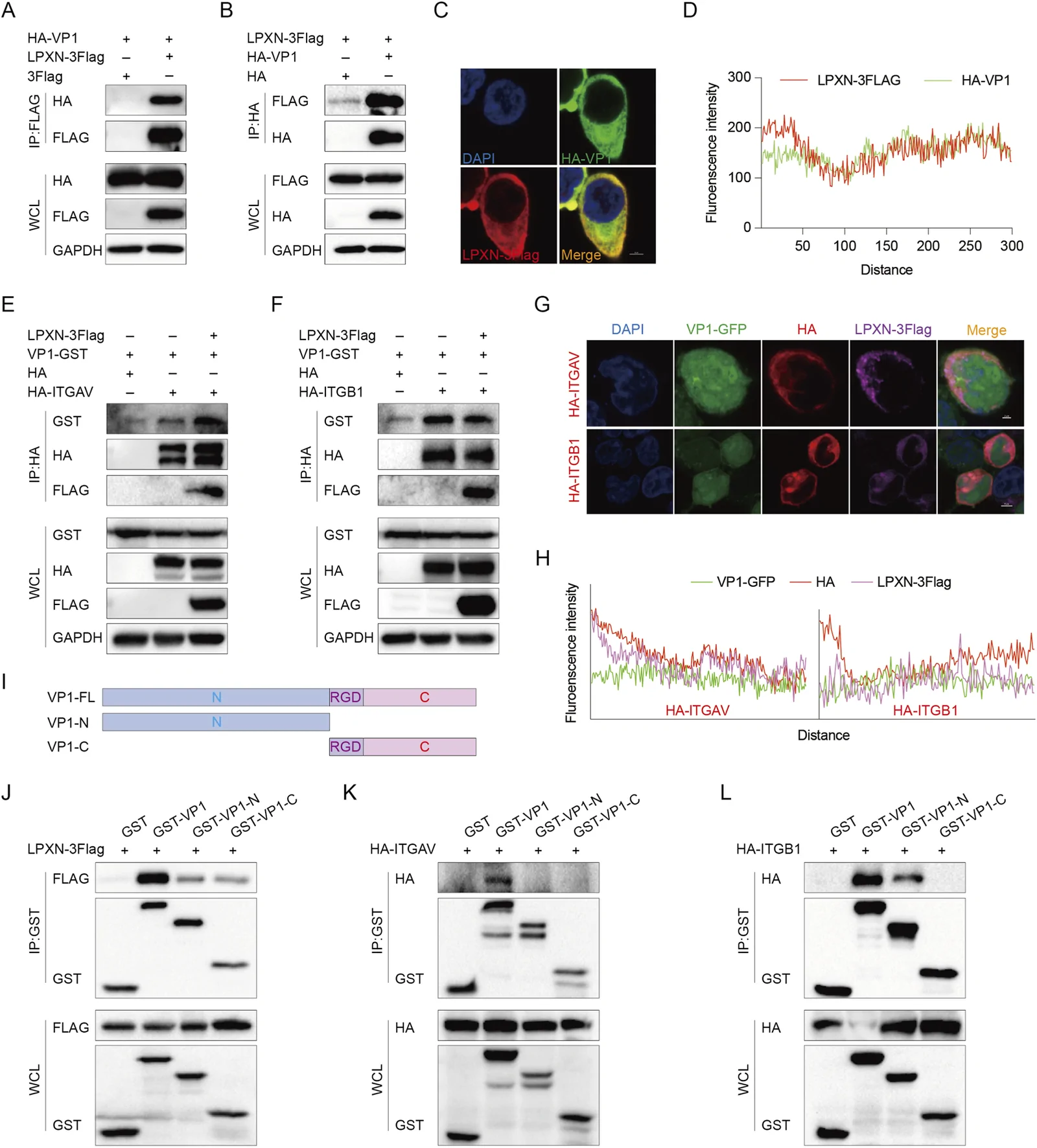
Ternary interaction among LPXN, VP1, and ITGAV/ITGB1. **(A,B)** Co-IP analysis in HEK293T cells co-transfected with LPXN-Flag and HA-VP1 for 24 h. **(C)** Confocal microscopy showing colocalization of LPXN-Flag (red) with HA-VP1 (green) in HEK293T cells. Scale bar: 5 µm. **(D)** Fluorescence intensity quantification at LPXN-VP1 colocalized sites. **(E,F)** Co-IP analysis in HEK293T cells expressing GST-VP1, HA-ITGAV **(E)**, or HA-ITGB1 **(F)**, with LPXN-Flag or empty vector for 24 h. **(G)** Confocal microscopy visualizing pairwise colocalization among HA-ITGAV (red)/HA-ITGB1 (red), VP1 (GFP-green), and LPXN-Flag (magenta) in HEK293T cells. Scale bars: 2 µm or 5 µm. **(H)** Fluorescence intensity quantification at sites of triple colocalization (LPXN, VP1, and ITGAV/ITGB1). **(I)** Schematic diagram of VP1 truncation mutants. **(J)** Co-IP analysis in HEK293T cells expressing LPXN-Flag with GST-VP1 (WT or truncated forms) or empty vector for 24 h. **(K, L)** Co-IP analysis in HEK293T cells expressing HA-ITGAV **(K)** or HA-ITGB1 **(L)** with GST-VP1 (WT or truncated forms) or empty vector for 24 h. All experiments were independently repeated at least three times (n = 3), with representative images/blots shown.

As LPXN is localized to the plasma membrane, interacts with and regulates the expression of ITGAV and ITGB1, and is involved in FMDV cellular entry, we further investigated its interaction with FMDV structural proteins, particularly the receptor-binding protein, VP1. Co-IP assays, including both forward and reverse immunoprecipitation, suggested the specific interaction between LPXN and VP1 (Figures 7A,B). Confocal microscopy further supported their colocalization at the plasma membrane (Figures 7C,D). Similar interactions were observed between LPXN and other FMDV structural proteins. Co-IP and immunofluorescence analyses showed that LPXN interacts with and colocalizes with VP2, VP3, and VP4 (Supplementary Figure S5A-C).

As both LPXN and FMDV VP1 are linked to integrin signaling, a key pathway for FMDV entry, we next investigated whether these three molecules assemble into a ternary complex. Using systematic Co-IP-based interaction mapping, we verified the formation of an integrin-LPXN-VP1 ternary complex. Co-IP assays showed strong interactions among integrin, LPXN, and VP1 (Figures 7E,F). Additionally, confocal microscopy after co-transfection with VP1-EGFP, LPXN-Flag, and HA-ITGAV/HA-ITGB1 plasmids into HEK293 T cells revealed clear colocalization of all three components at the plasma membrane (Figures 7G,H). To identify the specific domains mediating the interactions between LPXN, ITGAV/ITGB1, and VP1, we divided VP1 into N-terminal and C-terminal fragments using the RGD motif as the boundary (Figure 7I) (Burman et al., 2006; Fox et al., 1989). Co-IP assays showed that LPXN interacts with full-length VP1 and both its N- and C-terminal fragments (Figure 7J). In contrast, ITGAV interacted only with full-length VP1 (Figure 7K), whereas ITGB1 bound to full-length VP1 and its N-terminal fragment (Figure 7L). These findings suggest that LPXN, VP1, and either ITGAV or ITGB1 can assemble into a ternary complex.

**FIGURE 8:**
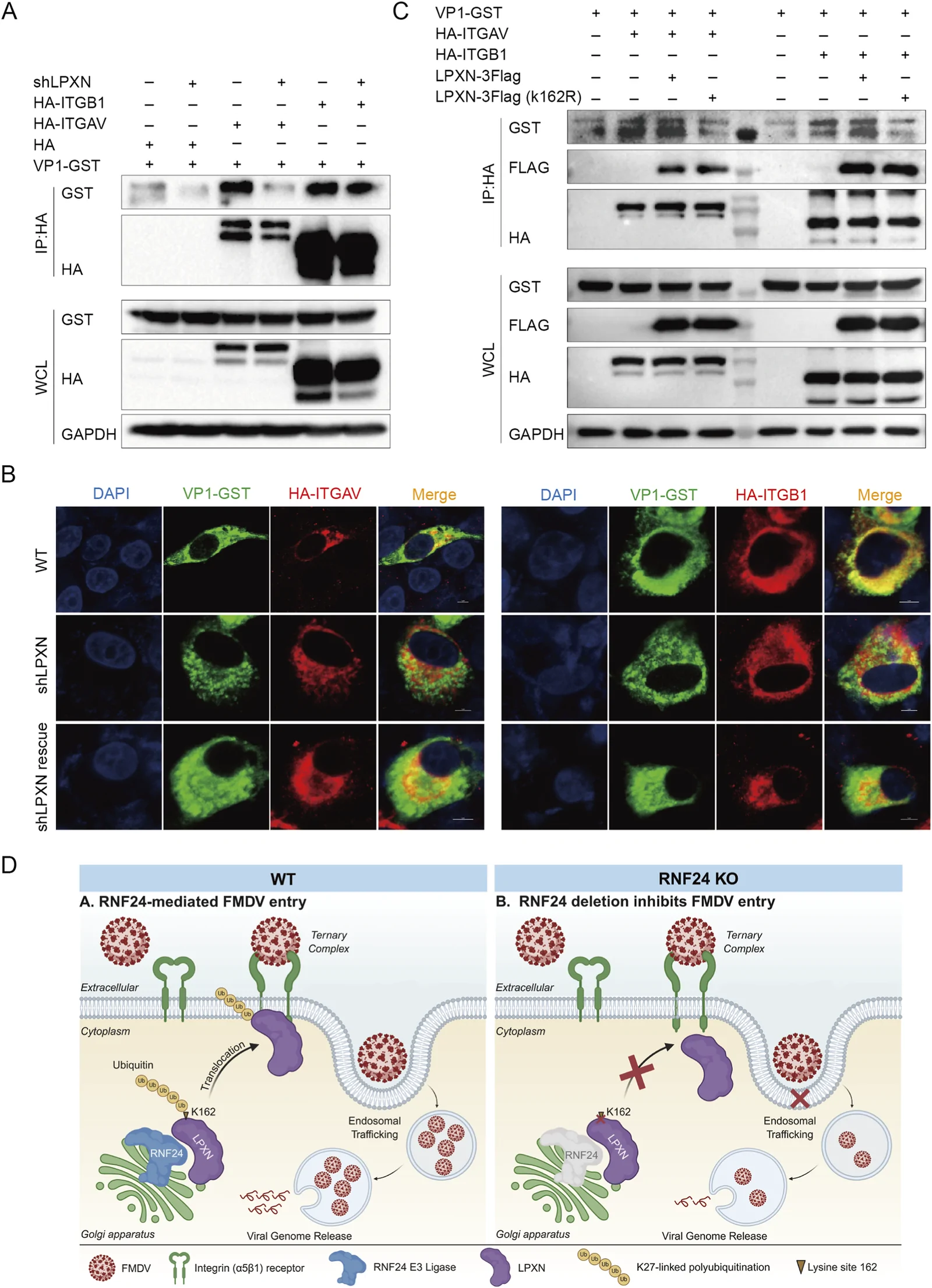
Lysine 162 of LPXN is critical for VP1-integrin interaction. **(A)** Co-IP analysis in shLPXN and shNC SK6 cells co-transfected with GST-VP1, HA-ITGAV/HA-ITGB1, or empty vector for 24 h. **(B)** Confocal microscopy showing colocalization of GST-VP1 (green) with HA-ITGAV or HA-ITGB1 (red) in shNC, shLPXN, and LPXN-rescued (LPXN-R) SK6 cells co-transfected with the indicated constructs for 24 h. Scale bar: 5 µm. **(C)** Co-IP analysis in HEK293T cells expressing GST-VP1, HA-ITGAV/HA-ITGB1, and Flag-LPXN (WT or K162R mutant) or empty vector for 24 h. **(D)** Proposed cellular model for RNF24 in FMDV entry. Left: RNF24 promotes K27-linked polyubiquitination of LPXN at K162, promoting its membrane translocation and scaffolding of an integrin-LPXN-VP1 ternary complex, which stabilizes virus-receptor binding and facilitates internalization. Right: Loss of RNF24 reduces membrane-localized LPXN, disrupts ternary complex assembly, and inhibits viral entry. All experiments were independently repeated at least three times (n = 3), with representative images/blots shown.

We further examined the effect of LPXN knockdown on the interaction between VP1 and ITGAV/ITGB1. Co-IP assays showed that the interaction between ITGAV and VP1 was significantly weakened in LPXN knockdown cells (Figure 8A). Confocal microscopy suggested that LPXN knockdown reduced the colocalization of VP1 with ITGAV and ITGB1, whereas LPXN complementation partially restored it (Figure 8B). These results indicate that LPXN acts as a scaffold connecting VP1 to integrins at the plasma membrane. Since RNF24 regulates LPXN through ubiquitination, we next examined whether the LPXN K162R mutant, which is defective in K27-linked ubiquitination, interacts normally with VP1, ITGAV, and ITGB1. The results showed that the K162R mutant failed to effectively promote VP1 binding to either ITGAV or ITGB1 (Figure 8C), indicating that RNF24-promoted K27-linked polyubiquitination of LPXN at K162 is essential for facilitating VP1-integrin interactions.

Together, these findings suggest that LPXN acts as a scaffold protein that promotes the assembly of a membrane-associated integrin-LPXN-VP1 ternary complex during FMDV infection. Importantly, RNF24-promoted K27-linked polyubiquitination of LPXN is required for its ability to link VP1 with ITGAV/ITGB1.

## Discussion

FMDV, a highly contagious pathogen, poses substantial economic challenges to the global livestock industry. However, numerous host factors critical for FMDV infection remain unidentified, and elucidating viral entry mechanisms is essential for designing effective antiviral interventions. In this study, we applied a porcine genome-wide CRISPR/Cas9 knockout screen and identified several host factors linked to FMDV infection, including *TINAG*, *HR, etc.* Notably, RNF24 was discovered for the first time as an essential host factor required for FMDV infection. Mechanistically, RNF24 facilitates the assembly of the integrin-VP1 complex by driving K27-linked non-degradative ubiquitination of LPXN. This central mechanism offers novel insights into FMDV entry (Figure 8D) and advances our comprehension of virus-host interactions.

Compared with a previously reported CRISPR knockout screen for FMDV (Peng et al., 2024), our study reveals key methodological and outcome distinctions. The earlier screen utilized a CRISPR library containing 93,859 sgRNAs targeting 16,886 protein-coding genes (Peng et al., 2024), whereas we employed the PigGeCKO v2.0 library (82,371 sgRNAs) covering 21,139 porcine protein-coding genes (Liu et al., 2025), providing broader genomic coverage for host factor discovery. Additionally, while the prior study used a single high multiplicity of infection (MOI = 0.2) for viral challenge, we performed three sequential screening rounds with progressively increasing MOIs (0.00001, 0.001, and 0.01) to enrich for more robust and functionally versatile host genes. Although both studies used an FMDV type O strain, our CRISPR screening was conducted in LLC-PK1 cells, a distinct FMDV-susceptible cell line, thereby complementing the earlier IBRS-2 cell line-based model. Notably, no overlap was observed among the top 30 candidate genes between the two CRISPR screens, demonstrating that distinct experimental designs can yield substantially different results. This comparison highlights that varying CRISPR screening conditions can uncover distinct sets of host factors for the same virus.

Notably, RNF24 was first identified in our CRISPR screen. Knockout of RNF24 impaired FMDV replication at an early stage of its life cycle. RNF24 localizes to the Golgi apparatus, a central hub for post-translational protein modification and vesicular trafficking (Bonifacino and Glick, 2004; Witkos and Lowe, 2017; Yadav and Linstedt, 2011), suggesting its potential involvement in viral replication or budding (Bergeman et al., 2024; Yin Y. C. et al., 2024). However, our results support that RNF24 regulates FMDV entry into host cells, thereby expanding the known mechanisms by which the Golgi apparatus influences viral entry. Moreover, we found that RNF24 promotes FMDV entry by modulating the integrin-mediated pathway. The role of the Golgi in regulating integrins is well established (Gee et al., 2018; Wan et al., 2014). Given that RNF24 interacts with both ITGAV and ITGB1, further investigation is warranted to determine whether RNF24 directly regulates integrins. Further exploring the role of RNF24 in FMDV infection in live animals represents a promising direction for future investigation.

RNF24 is a member of the RING finger protein family, which includes numerous E3 ubiquitin ligases (Cai et al., 2022); however, its ubiquitination activity has not been reported to date. In this study, we show that RNF24 promotes K27-linked ubiquitination of LPXN at the K162 site, thereby establishing its E3 ubiquitin ligase function for the first time. K27-linked ubiquitination is generally non-degradative and known to modulate several cellular signaling pathways (Chen et al., 2024; Gatti et al., 2015; Liu et al., 2014; Wang et al., 2021; Zhao et al., 2021). Our findings reveal that this site-specific modification contributes to LPXN membrane translocation, thereby influencing FMDV entry and further expanding the mechanistic understanding of non-degradative ubiquitination in viral infection. A limitation of the present study is that the mechanistic experiments were performed mainly in porcine cell lines. Whether the RNF24-LPXN axis operates in bovine and ovine cells, as well as *in vivo* during FMDV infection of natural hosts, remains to be determined and will be an important direction for future work. Notably, while LPXN’s paralog paxillin (PXN) has been implicated in influenza virus (IAV) entry (Guo et al., 2025), the role of LPXN itself in viral infections has remained entirely unknown. Our work newly identifies LPXN as a key regulator of FMDV entry, suggesting that the paxillin protein family may play evolutionarily conserved roles in mediating viral infection.

Moreover, our findings indicate that LPXN acts as a scaffold protein to facilitate the assembly of an integrin-LPXN-VP1 ternary complex on the cell membrane. This complex stabilizes the interaction between FMDV VP1 and host integrins, thereby promoting viral adsorption and entry. This result suggests that FMDV entry is not exclusively mediated by HSPGs and integrins, but likely involves additional host membrane proteins that cooperate in regulating the process. This may explain why deletion of individual known receptors does not completely block FMDV entry. In addition, RNF24-promoted K27-linked ubiquitination of LPXN is required for ternary complex formation, as the LPXN K162R mutant disrupts this assembly. Such precise regulation highlights the sophisticated interplay among host proteins and the complexity of virus-host interactions. Nevertheless, the dynamic regulatory mechanisms governing ternary complex assembly remain unclear and warrant further investigation using live-cell imaging approaches.

RNF24 knockout reduced ITGAV and ITGB1 mRNA and protein levels, whereas RNF24 overexpression did not affect integrin expression. This suggests that the pro-viral effect of RNF24 overexpression is likely mediated indirectly through LPXN, consistent with the observation that RNF24 enhances VP1-integrin association without increasing total integrin abundance. We therefore propose that RNF24 promotes FMDV entry primarily through LPXN ubiquitination and LPXN-dependent membrane scaffolding of the VP1-integrin complex, although RNF24-dependent changes in integrin expression may also contribute in knockout cells. The mechanisms by which RNF24 knockout affects integrin expression, whether transcriptional, post-transcriptional, or LPXN-dependent trafficking, remain to be further explored.

Our findings establish RNF24 as a previously uncharacterized proviral RNF family member with a distinct mechanism. Unlike many c-family E3 ligases that exert antiviral effects via viral protein degradation or innate immune signaling (Zhang et al., 2025; Fan et al., 2016; Yin et al., 2025; Xiong et al., 2020), RNF24 enhances FMDV entry by promoting non-degradative K27-linked ubiquitination of LPXN, which regulates its membrane scaffolding function rather than targeting it for proteasomal degradation. This ubiquitin-dependent scaffolding mechanism represents a novel mode of action for RNF-family proteins in virus-host interactions. The incomplete inhibition observed after RNF24 depletion is likely explained by the redundant and multifactorial nature of FMDV entry, in which multiple integrins, HSPGs, and additional host cofactors may compensate partially for loss of the RNF24-LPXN pathway. Because multiple viruses use integrins or integrin-associated signaling platforms during attachment and uptake, the RNF24-LPXN pathway may represent a broader host mechanism that modulates membrane receptor-complex assembly. However, the present data are limited to FMDV, and whether RNF24-LPXN is also exploited by adenoviruses, enteroviruses, coronaviruses, or other integrin-dependent viruses requires direct comparative infection studies.

## Conclusion

In summary, this study delineates a regulatory axis through which RNF24 facilitates FMDV entry by promoting K27-linked ubiquitination of LPXN at the K162 residue, thereby facilitating the assembly of the integrin-LPXN-VP1 complex. Our findings identify RNF24 and LPXN as key host regulators of FMDV infection, extend the current understanding of integrin-mediated viral entry, and provide deeper insights into the FMDV-host interaction network. This work highlights a potential antiviral target against FMDV, offers theoretical guidance for optimizing prevention strategies, and lays a foundation for developing host-directed antiviral approaches.

## Data Availability

The original contributions presented in the study are included in the article/Supplementary Material, further inquiries can be directed to the corresponding authors.

## References

[B1] Bellucci M. A. Amiri M. Berryman S. Moshari A. Owino C. O. Luteijn R. D. (2025). ITAF_45_ is a pervasive *trans*-acting factor for picornavirus type II IRES elements. P Natl. Acad. Sci. U. S. A. 122, e2506281122. 10.1073/pnas.2506281122 40789037 PMC12377779

[B2] Bergeman M. H. Velarde K. Hargis H. L. Glenn H. L. Hogue I. B. (2024). The Rab6 post-golgi secretory pathway contributes to Herpes simplex virus 1 (HSV-1) egress. J. Virol. 98, e0059924. 10.1128/jvi.00599-24 39136459 PMC11406995

[B3] Bonifacino J. S. Glick B. S. (2004). The mechanisms of vesicle budding and fusion. Cell 116, 153–166. 10.1016/s0092-8674(03)01079-1 14744428

[B4] Burman A. Clark S. Abrescia N. G. A. Fry E. E. Stuart D. I. Jackson T. (2006). Specificity of the VP1 GH loop of foot-and-mouth disease virus for αv integrins. J. Virol. 80, 9798–9810. 10.1128/JVI.00577-06 16973584 PMC1617245

[B5] Cabezas A. H. Sanderson M. W. Jaberi-Douraki M. Volkova V. V. (2018). Clinical and infection dynamics of foot-and-mouth disease in beef feedlot cattle: an expert survey. Prev. Vet. Med. 158, 160–168. 10.1016/j.prevetmed.2018.08.007 30220390

[B6] Cai C. M. Tang Y. D. Zhai J. B. Zheng C. F. (2022). The RING finger protein family in health and disease. Signal Transduct. Tar 7, 300. 10.1038/s41392-022-01152-2 36042206 PMC9424811

[B7] Chen M. R. Zhang X. Kong F. S. Gao P. Ge X. N. Zhou L. (2024). Senecavirus A induces mitophagy to promote self-replication through direct interaction of 2C protein with K27-linked ubiquitinated TUFM catalyzed by RNF185. Autophagy 20, 1286–1313. 10.1080/15548627.2023.2293442 38084826 PMC11210902

[B8] Dejarnac O. Hafirassou M. L. Chazal M. Versapuech M. Gaillard J. Perera-Lecoin M. (2018). TIM-1 ubiquitination mediates dengue virus entry. Cell Rep. 23, 1779–1793. 10.1016/j.celrep.2018.04.013 29742433

[B9] Dikic I. (2017). Proteasomal and autophagic degradation systems. Annu. Rev. Biochem. 86, 193–224. 10.1146/annurev-biochem-061516-044908 28460188

[B10] Fan W. C. Zhang D. Qian P. Qian S. H. Wu M. G. Chen H. C. (2016). Swine TRIM21 restricts FMDV infection via an intracellular neutralization mechanism. Antivir. Res. 127, 32–40. 10.1016/j.antiviral.2016.01.004 26777733

[B11] Fox G. Parry N. R. Barnett P. V. Mcginn B. Rowlands D. J. Brown F. (1989). The cell attachment site on foot-and-mouth-disease virus includes the amino-acid sequence rgd (arginine-glycine-aspartic acid). J. Gen. Virol. 70, 625–637. 10.1099/0022-1317-70-3-625 2543752

[B12] Gatti M. Pinato S. Maiolica A. Rocchio F. Prato M. G. Aebersold R. (2015). RNF168 promotes noncanonical K27 ubiquitination to signal DNA damage. Cell Rep. 10, 226–238. 10.1016/j.celrep.2014.12.021 25578731

[B13] Gee H. Y. Kim J. Lee M. G. (2018). Unconventional secretion of transmembrane proteins. FSemin Cell Dev. Biol. 83, 59–66. 10.1016/j.semcdb.2018.03.016 29580969

[B14] Grubman M. J. Baxt B. (2004). Foot-and-mouth disease. Clin. Microbiol. Rev. 17, 465–493. 10.1128/cmr.17.2.465-493.2004 15084510 PMC387408

[B15] Guo J. M. Zhou Z. Li R. N. Xing Z. F. Zhang L. Zhao S. Y. (2025). A genome-wide base-editing screen uncovers a pivotal role of paxillin δ ubiquitination in influenza virus infection. Cell Rep. 44, 115748. 10.1016/j.celrep.2025.115748 40434888

[B16] Gupta A. Lee B. S. Khadeer M. A. Tang Z. H. Chellaiah M. Abu-Amer Y. (2003). Leupaxin is a critical adaptor protein in the adhesion zone of the osteoclast. J. Bone Min. Res. 18, 669–685. 10.1359/jbmr.2003.18.4.669 12674328

[B17] Hu S. J. Gan M. L. Wei Z. Shang P. Song L. Feng J. K. (2024). Identification of host factors for livestock and poultry viruses: genome-wide screening technology based on the CRISPR system. Front. Microbiol. 15, 1498641. 10.3389/fmicb.2024.1498641 39640855 PMC11619636

[B18] Jackson T. Ellard F. M. Ghazaleh R. A. Brookes S. M. Blakemore W. E. Corteyn A. H. (1996). Efficient infection of cells in culture by type O foot-and-mouth disease virus requires binding to cell surface heparan sulfate. J. Virol. 70, 5282–5287. 10.1128/JVI.70.8.5282-5287.1996 8764038 PMC190485

[B19] Jackson T. Sheppard D. Denyer M. Blakemore W. King A. M. Q. (2000). The epithelial integrin αvβ6 is a receptor for foot-and-mouth disease virus. J. Virol. 74, 4949–4956. 10.1128/jvi.74.11.4949-4956.2000 10799568 PMC110846

[B20] Jackson T. Mould A. P. Sheppard D. King A. M. Q. (2002). Integrin αvβ1 is a receptor for foot-and-mouth disease virus. J. Virol. 76, 935–941. 10.1128/jvi.76.3.935-941.2002 11773368 PMC135819

[B21] Jackson T. Clark S. Berryman S. Burman A. Cambier S. Mu D. Z. (2004). Integrin αvβ8 functions as a receptor for foot-and-mouth disease virus: role of the β-chain cytodomain in integrin-mediated infection. J. Virol. 78, 4533–4540. 10.1128/jvi.78.9.4533-4540.2004 15078934 PMC387692

[B22] Jamal S. M. Belsham G. J. (2013). Foot-and-mouth disease: past, present and future. Vet. Res. 44, 116. 10.1186/1297-9716-44-116 24308718 PMC4028749

[B23] Kabadi A. M. Ousterout D. G. Hilton I. B. Gersbach C. A. (2014). Multiplex CRISPR/Cas9-based genome engineering from a single lentiviral vector. Nucleic Acids Res. 42, e147. 10.1093/nar/gku749 25122746 PMC4231726

[B24] Kim H. S. Lee K. Bae S. Park J. Lee C. K. Kim M. (2017). CRISPR/Cas9-mediated gene knockout screens and target identification via whole-genome sequencing uncover host genes required for picornavirus infection. J. Biol. Chem. 292, 10664–10671. 10.1074/jbc.M117.782425 28446605 PMC5481571

[B25] Kitching R. P. (2002). Clinical variation in foot and mouth disease: cattle. Rev. Sci. Tech. Oie 21, 499–504. 10.20506/rst.21.3.1343 12523690

[B26] Klapproth S. Bromberger T. Türk C. Krüger M. Moser M. (2019). A kindlin-3-leupaxin-paxillin signaling pathway regulates podosome stability. J. Cell Biol. 218, 3436–3454. 10.1083/jcb.201903109 31537712 PMC6781449

[B27] Klöhn M. Burkard T. Janzen J. Haase J. A. Gömer A. Fu R. (2024). Targeting cellular cathepsins inhibits hepatitis E virus entry. Hepatology 80, 1239–1251. 10.1097/hep.0000000000000912 38728662 PMC11486972

[B28] Li K. L. Wang C. C. Yang F. Cao W. J. Zhu Z. X. Zheng H. X. (2021). Virus-host interactions in foot-and-mouth disease virus infection. Front. Immunol. 12, 571509. 10.3389/fimmu.2021.571509 33717061 PMC7952751

[B29] Liang Y. S. Zhang G. G. Li Q. H. Han L. Hu X. Y. Guo Y. (2021). TRIM26 is a critical host factor for HCV replication and contributes to host tropism. Sci. Adv. 7, eabd9732. 10.1126/sciadv.abd9732 33523994 PMC7793585

[B30] Liu S. Thomas S. M. Woodside D. G. Rose D. M. Kiosses W. B. Pfaff M. (1999). Binding of paxillin to α4 integrins modifies integrin-dependent biological responses. Nature 402, 676–681. 10.1038/45264 10604475

[B31] Liu J. Han C. F. Xie B. Wu Y. Liu S. X. Chen K. (2014). Rhbdd3 controls autoimmunity by suppressing the production of IL-6 by dendritic cells via K27-linked ubiquitination of the regulator NEMO. Nat. Immunol. 15, 612–622. 10.1038/ni.2898 24859449

[B32] Liu H. S. Xue Q. Yang F. Cao W. J. Liu P. F. Liu X. T. (2024). Foot-and-mouth disease virus VP1 degrades YTHDF2 through autophagy to regulate IRF3 activity for viral replication. Autophagy 20, 1597–1615. 10.1080/15548627.2024.2330105 38516932 PMC11210904

[B33] Liu H. L. Guo Y. L. Guo Z. S. Sun L. M. Tao D. G. Li X. Y. (2025). Integration of CRISPR screening and proteomic analysis of WDR91 manipulation of endosome-to-cytosol transport of African swine fever virus. Sci. China Life Sci. 68, 1839–1842. 10.1007/s11427-024-2739-9 39960627

[B34] Lussier M. P. Lepage P. K. Bousquet S. M. Boulay G. (2008). RNF24, a new TRPC interacting protein, causes the intracellular retention of TRPC. Cell Calcium 43, 432–443. 10.1016/j.ceca.2007.07.009 17850865

[B35] Lv B. N. Yuan Y. C. Yang Z. Wang X. R. Hu J. J. Sun Y. D. (2024). Stearoyl coenzyme A desaturase 1 (SCD1) regulates foot-and-mouth disease virus replication by modulating host cell lipid metabolism and viral protein 2C-mediated replication complex formation. J. Virol. 98, e0090224. 10.1128/jvi.00902-24 39324793 PMC11495015

[B36] Ma K. Hao R. Z. Liu T. R. Ru Y. Feng T. Qi X. L. (2025). Prolyl endopeptidase is a multifunctional host factor required for FMDV infection. Cell Mol. Life Sci. 82, 408. 10.1007/s00018-025-05941-0 41258568 PMC12630515

[B37] Marceau C. D. Puschnik A. S. Majzoub K. Ooi Y. S. Brewer S. M. Fuchs G. (2016). Genetic dissection of host factors through genome-scale CRISPR screens. Nature 535, 159–163. 10.1038/nature18631 27383987 PMC4964798

[B38] Mercer J. Schelhaas M. Helenius A. (2010). Virus entry by endocytosis. Annu. Rev. Biochem. 79, 803–833. 10.1146/annurev-biochem-060208-104626 20196649

[B39] Neff S. Sá-Carvalho D. Rieder E. Mason P. W. Blystone S. D. Brown E. J. (1998). Foot-and-mouth disease virus virulent for cattle utilizes the integrin alpha(v)beta3 as its receptor. J. Virol. 72, 3587–3594. 10.1128/JVI.72.5.3587-3594.1998 9557639 PMC109579

[B40] Peng J. L. Yi J. M. Yang W. P. Ren J. J. Wen Y. Zheng H. X. (2020). Advances in foot-and-mouth disease virus proteins regulating host innate immunity. Front. Microbiol. 11, 2046. 10.3389/fmicb.2020.02046 33162944 PMC7581685

[B41] Peng G. C. Liu T. R. Qi X. L. Wang Y. Z. Ren J. J. Peng J. L. (2024). A genome-wide CRISPR screening uncovers that TOB1 acts as a key host factor for FMDV infection via both IFN and EGFR mediated pathways. Plos Pathog. 20, e1012104. 10.1371/journal.ppat.1012104 38512977 PMC10986976

[B42] Poonsuk K. Giménez-Lirola L. Zimmerman J. J. (2018). A review of foot-and-mouth disease virus (FMDV) testing in livestock with an emphasis on the use of alternative diagnostic specimens. Anim. Health Res. Rev. 19, 100–112. 10.1017/S1466252318000063 30345947

[B43] Pulido M. R. Sáiz M. (2017). Molecular mechanisms of foot-and-mouth disease virus targeting the host antiviral response. Front. Cell Infect. Mi 7, 252. 10.3389/fcimb.2017.00252 PMC5468379 28660175

[B44] Qiao W. J. Richards C. M. Kim Y. Zengel J. R. Ding S. Y. Greenberg H. B. (2024). MYADM binds human parechovirus 1 and is essential for viral entry. Nat. Commun. 15, 3469. 10.1038/s41467-024-47825-0 38658526 PMC11043367

[B45] Qiao W. J. Xie X. P. Shi P. Y. Ooi Y. S. Carette J. E. (2025). Druggable genome screens identify SPP as an antiviral host target for multiple flaviviruses. P Natl. Acad. Sci. U. S. A. 122, e2421573122. 10.1073/pnas.2421573122 PMC11874179 39969998

[B46] Seago J. Juleff N. Moffat K. Berryman S. Christie J. M. Charleston B. (2013). An infectious recombinant foot-and-mouth disease virus expressing a fluorescent marker protein. J. Gen. Virol. 94, 1517–1527. 10.1099/vir.0.052308-0 23559477 PMC3709630

[B47] Stenfeldt C. Eschbaumer M. Humphreys J. Medina G. N. Arzt J. (2025). The pathogenesis of foot-and-mouth disease virus: current understandings and knowledge gaps. Vet. Res. 56, 119. 10.1186/s13567-025-01545-5 40524230 PMC12172366

[B48] Strich J. R. Chertow D. S. (2019). CRISPR-cas biology and its application to infectious diseases. J. Clin. Microbiol. 57, e01307-18. 10.1128/JCM.01307-18 30429256 PMC6440769

[B49] Sundberg-Smith L. J. DiMichele L. A. Sayers R. L. Mack C. P. Taylor J. M. (2008). The LIM protein leupaxin is enriched in smooth muscle and functions as an serum response factor cofactor to induce smooth muscle cell gene transcription. Circ. Res. 102, 1502–1511. 10.1161/CIRCRESAHA.107.170357 18497331 PMC2785029

[B50] Tanaka T. Moriwaki K. Murata S. Miyasaka M. (2010). LIM domain-containing adaptor, leupaxin, localizes in focal adhesion and suppresses the integrin-induced tyrosine phosphorylation of paxillin. Cancer Sci. 101, 363–368. 10.1111/j.1349-7006.2009.01398.x 19917054 PMC11158308

[B51] Tang J. L. Abdullah S. W. Li P. H. Wu J. E. Pei C. C. Mu S. Y. (2022). Heat shock protein 60 is involved in viral replication complex formation and facilitates foot and mouth virus replication by stabilizing viral nonstructural proteins 3A and 2C. Mbio 13, e0143422. 10.1128/mbio.01434-22 36106732 PMC9601101

[B52] Wallace L. E. de Vries E. van Kuppeveld F. J. M. de Haan C. A. M. (2023). Neuraminidase-dependent entry of influenza A virus is determined by hemagglutinin receptor-binding specificity. J. Virol. 97, e0060223. 10.1128/jvi.00602-23 37754760 PMC10617504

[B53] Wan J. Zhu F. Zasadi L. M. Yu J. Q. Wang L. Johnson A. (2014). A golgi-localized pool of the mitotic checkpoint component Mad1 controls integrin secretion and cell migration. Curr. Biol. 24, 2687–2692. 10.1016/j.cub.2014.09.052 25447996 PMC4254593

[B54] Wang G. X. Wang Y. H. Shang Y. J. Zhang Z. D. Liu X. T. (2015). How foot-and-mouth disease virus receptor mediates foot-and-mouth disease virus infection. Virol. J. 12, 9. 10.1186/s12985-015-0246-z 25645358 PMC4322448

[B55] Wang B. B. Wang M. Zhang W. B. Xiao T. F. Chen C. H. Wu A. (2019). Integrative analysis of pooled CRISPR genetic screens using MAGeCKFlute. Nat. Protoc. 14, 756–780. 10.1038/s41596-018-0113-7 30710114 PMC6862721

[B56] Wang X. E. Zhang H. H. Shao Z. G. Zhuang W. X. Sui C. Liu F. (2021). TRIM31 facilitates K27-linked polyubiquitination of SYK to regulate antifungal immunity. Signal Transduct. Tar 6, 298. 10.1038/s41392-021-00711-3 34362877 PMC8342987

[B57] Wang X. H. Hu Z. L. Zhang W. Wu S. W. Hao Y. J. Xiao X. (2023). Inhibition of lysosome-tethered Ragulator-Rag-3D complex restricts the replication of enterovirus 71 and coxsackie A16. J. Cell Biol. 222, e202303108. 10.1083/jcb.202303108 37906052 PMC10619577

[B58] Wang T. T. Jiang J. Zhang X. Ke X. S. Qu Y. (2024). Ubiquitin-like modification dependent proteasomal degradation and disease therapy. Trends Mol. Med. 30, 1061–1075. 10.1016/j.molmed.2024.05.005 38851992

[B59] Witkos T. M. Lowe M. (2017). Recognition and tethering of transport vesicles at the golgi apparatus. Curr. Opin. Cell Biol. 47, 16–23. 10.1016/j.ceb.2017.02.003 28237810

[B60] Xiong M. G. Xu Z. S. Li Y. H. Wang S. Y. Wang Y. Y. Ran Y. (2020). RNF152 positively regulates TLR/IL-1R signaling by enhancing MyD88 oligomerization. EMBO Rep. 21, e48860. 10.15252/embr.201948860 31930677 PMC7054668

[B61] Yadav S. Linstedt A. D. (2011). Golgi positioning. Csh Perspect. Biol. 3, a005322. 10.1101/cshperspect.a005322 21504874 PMC3101843

[B62] Yang P. Yuan Y. C. Sun Y. D. Lv B. N. Du H. Zhou Z. (2023). The host protein CAD regulates the replication of FMDV through the function of pyrimidines' synthesis. J. Virol. 97, e0036923. 10.1128/jvi.00369-23 37162335 PMC10231220

[B63] Yin M. G. Qian P. Wang H. Y. Zhao Q. Q. Zhang H. Y. Zheng Z. X. (2024a). Foot-and-mouth disease virus (FMDV) negatively regulates ZFP36 protein expression to alleviate its antiviral activity. J. Virol. 98, e0111424. 10.1128/jvi.01114-24 39194213 PMC11406947

[B64] Yin Y. C. Kan X. J. Miao X. Y. Sun Y. J. Chen S. J. Qin T. (2024b). H5 subtype Avian influenza virus induces golgi apparatus stress response *via* TFE3 pathway to promote virus replication. Plos Pathog. 20, e1012748. 10.1371/journal.ppat.1012748 39652582 PMC11627363

[B65] Yin M. G. Li X. M. Zhang M. Zhao Q. Q. Wang H. Y. Zhang H. Y. (2025). MARCH8 promotes the proteasomal degradation of foot-and-mouth disease virus VP1, VP2, and VP3 to negatively regulate viral replication. Vet. Res. 56, 96. 10.1186/s13567-025-01521-z 40307900 PMC12044826

[B66] Zajac N. Vlachos I. S. Sajibu S. Opitz L. Wang S. S. Chittur S. V. (2025). The impact of PCR duplication on RNAseq data generated using NovaSeq 6000, NovaSeq X, AVITI, and G4 sequencers. Genome Biol. 26, 145. 10.1186/s13059-025-03613-7 40437619 PMC12117910

[B67] Zhang R. Miner J. J. Gorman M. J. Rausch K. Ramage H. White J. P. (2016). A CRISPR screen defines a signal peptide processing pathway required by flaviviruses. Nature 535, 164–168. 10.1038/nature18625 27383988 PMC4945490

[B68] Zhang H. J. Wang X. W. Qu M. Li Z. Y. Yin X. P. Tang L. J. (2023). Foot-and-mouth disease virus structural protein VP3 interacts with HDAC8 and promotes its autophagic degradation to facilitate viral replication. Autophagy 19, 2869–2883. 10.1080/15548627.2023.2233847 37408174 PMC10549200

[B69] Zhang W. Li W. W. Yang Y. Cao W. J. Shao W. H. Huang M. Y. (2025). RING finger protein 5 is a key anti-FMDV host factor through inhibition of virion assembly. Plos Pathog. 21, e1012848. 10.1371/journal.ppat.1012848 39823440 PMC11741381

[B70] Zhao C. Z. Liu H. L. Xiao T. H. Wang Z. C. Nie X. W. Li X. Y. (2020). CRISPR screening of porcine sgRNA library identifies host factors associated with Japanese encephalitis virus replication. Nat. Commun. 11, 5178. 10.1038/s41467-020-18936-1 33057066 PMC7560704

[B71] Zhao D. S. Zhong G. H. Li J. W. Pan J. J. Zhao Y. L. Song H. L. (2021). Targeting E3 ubiquitin ligase WWP1 prevents cardiac hypertrophy through destabilizing DVL2 via inhibition of K27-Linked ubiquitination. Circulation 144, 694–711. 10.1161/circulationaha.121.054827 34139860

