# Supplementary material for: Genome-wide CRISPR screen identifies RNF24 as a critical host factor for foot-and-mouth disease virus entry
Source: Front Cell Dev Biol. 2026 Aug 19;14:1909904. doi: 10.3389/fcell.2026.1909904 (PMC13533954; doi:10.3389/fcell.2026.1909904)
Supplement: Supplementary file 1 [file DataSheet1.docx]

**SUPPLEMENTAL MATERIAL**

**Genome-Wide CRISPR Screen Identifies RNF24 as a Critical Host Factor for Foot-and-Mouth Disease Virus Entry**

Jinyan Zhang^1,2,4,#^, Hailong Liu^4,6,#^, Jian Du^1,2,4,#^, Min Zhang^1,2,4^, Qiongqiong Zhao^1,2,4^, Mengge Yin^1,2,4^, Xinghua Chen^1,2,4^, Xiangmin Li^1,2,3,4,5^, Zengjun Lu^7*^, Shengsong Xie^4,5,6*^, Ping Qian^1,2,3,4,5*^

Ping Qian

Email: [qianp@mail.hzau.edu.cn](mailto:qianp@mail.hzau.edu.cn)

**This file includes:**

Figures S1 to S5

Tables S2 to S6

**Figure S1. Cytopathic effects in FMDV-infected LLC-PK1-Cas9 cells at varying MOIs.**
LLC-PK1-Cas9 cells were infected with FMDV at different MOIs (0.01, 0.001, and 0.00001). Cytopathic effects (CPE) were observed and recorded at 24, 36, and 48 hours post‑infection (hpi). Scale bar: 200 µm.

Representative images are shown from three independent experiments (n = 3).

**Figure S2. LPXN interacts with ITGAV and ITGB1 and regulates their expression.**
(A, B) Western blot analysis of ITGAV (A) and ITGB1 (B) protein levels in LPXN-overexpressing SK6 cells with or without FMDV infection.
(C, D) Co-IP analysis in HEK293T cells co-transfected with HA-ITGAV/HA-ITGB1 and LPXN-Flag or empty vector for 24 h.
(E) Co-IP analysis in HEK293T cells co-transfected with LPXN-Flag and HA-ITGAV/HA-ITGB1 or empty vector for 24 h.
(F) Confocal microscopy showing colocalization of LPXN-Flag (red) with HA-ITGAV or HA-ITGB1 (green) in HEK293T cells. Scale bar: 5 µm.
(G) Quantification of fluorescence intensity at LPXN-ITGAV and LPXN-ITGB1 colocalized sites.

All experiments were independently repeated at least three times (n = 3), with representative images/blots shown.

**Figure S3. RNF24 promotes LPXN ubiquitination and affects FMDV replication via its E3 ubiquitin ligase activity.**

(A) Co-IP analysis in HEK293T cells co-transfected with Myc-Ub, HA-LPXN, and Flag-RNF24, or the indicated Flag-RNF24 RING-domain mutant (H98A/H101A and C78A/C81A/H98A/H101A) for 24 h.

(B) Co-IP analysis in RNF24-KO versus WT cells co-transfected with Myc-Ub, HA-LPXN, and Flag-RNF24, or the indicated Flag-RNF24 RING-domain mutant (H98A/H101A and C78A/C81A/H98A/H101A) for 24 h.

(C) VP1 expression detected by Western blot in LLC-PK1 cells transfected with Flag-RNF24-WT, Flag-RNF24-H98A/H101A, or Flag-RNF24-C78A/C81A/H98A/H101A at 12 h post-infection with FMDV (MOI = 0.1).

All experiments were independently repeated at least three times (n = 3), with representative blots shown.

**Figure S4. RNF24 mediates ubiquitination within the LIM1 and LIM2 domains of LPXN.**

(A) Co-IP analysis in HEK293T cells expressing HA-Ub, LPXN-LD-Flag or LPXN-LIM-Flag (truncated forms), and Myc-RNF24 or empty vector for 24 h.

(B, C) Co-IP analysis in HEK293T cells expressing HA-Ub, LPXN-ΔLIM3+4 -Flag (B) or LPXN-ΔLIM3+4-Flag (C) (truncated forms), and Myc-RNF24 or empty vector for 24 h.

All experiments were independently repeated at least three times (n = 3), with representative blots shown.

**Figure S5. LPXN interacts with VP1, VP2, VP3 and VP4.**
(A) Co-IP analysis in HEK293T cells co‑expressing GST-tagged VP1, VP2, VP3, or VP4 with LPXN‑Flag or empty vector for 24 h.
(B) Co-IP analysis in HEK293T cells co-expressing LPXN-Flag with GST-tagged VP1, VP2, VP3, VP4, or empty vector for 24 h.
(C) Confocal microscopy showing colocalization of LPXN-Flag (green) with HA-VP2, HA-VP3, or HA-VP4 (red) in HEK293T cells. Scale bar: 5 µm.

All experiments were independently repeated at least three times (n = 3), with representative images/blots shown.

**Table S2 Ubiquitination sites of LPXN identified by mass spectrometry**

| **Position** | **Amino acid** | **Modified sequence** | **Confidence score** |
| --- | --- | --- | --- |
| 36 | K | _K[GlyGly (K)]ESDLAETSK_ | 1 |
| 79 | K | _VYSEVQEPK[GlyGly (K)]ESPPPPK_ | 1 |
| 86 | K | _ESPPPPK[GlyGly (K)]TSAAAQLDELM[Oxidation (M)]AHLC[Carbamidomethyl (C)]EMQIQGTVK_ | 1 |
| 116 | K | _VDASTK[GlyGly (K)]HLPEK_ | 1 |
| 121 | K | _HLPEK[GlyGly (K)]QDPK_ | 1 |
| 125 | K | _HLPEK[GlyGly (K)]QDPK[GlyGly (K)]TSLDSM[Oxidation (M)]LGGLEQELQDLGIATVPK_ | 1 |
| 157 | K | _GHC[Carbamidomethyl (C)]ASC[Carbamidomethyl (C)]QK[GlyGly (K)]PIAGK_ | 1 |
| 162 | K | _GHC[Carbamidomethyl (C)]ASC[Carbamidomethyl (C)]QKPIAGK[GlyGly (K)]MIHALGQAWHPEHFVC[Carbamidomethyl (C)]AHC[Carbamidomethyl (C)]K_ | 0.99999994 |
| 182 | K | _MIHALGQAWHPEHFVC[Carbamidomethyl (C)]AHC[Carbamidomethyl (C)]K[GlyGly (K)]EEIGSSPFFER_ | 1 |
| 201 | K | _TGLAYC[Carbamidomethyl (C)]SK[GlyGly (K)]DYHHLFSPR_ | 1 |
| 252 | K | _VLTAMDQTWHPEHFFC[Carbamidomethyl (C)]AHC[Carbamidomethyl (C)]GEVFGAEGFHEK[GlyGly (K)]DK_ | 1 |
| 254 | K | _DK[GlyGly (K)]KPYC[Carbamidomethyl (C)]R_ | 1 |
| 260 | K | _K[GlyGly (K)]DFLAMFSPK_ | 1 |
| 269 | K | _DFLAMFSPK[GlyGly (K)]C[Carbamidomethyl (C)]GGC[Carbamidomethyl (C)]NR_ | 1 |
| 364 | K | _FHPEHFVC[Carbamidomethyl (C)]DFC[Carbamidomethyl (C)]LMQLSK[GlyGly (K)]GIFR_ | 1 |
| 373 | K | _EQNDK[GlyGly (K)]TYC[Carbamidomethyl (C)]QPC[Carbamidomethyl (C)]FNK_ | 1 |
| 382 | K | _TYC[Carbamidomethyl (C)]QPC[Carbamidomethyl (C)]FNK[GlyGly (K)]LFTQ_ | 1 |

**Table S3 Primers used in this study**

| **Primers ID** | **Sequences (5’-3’)** |
| --- | --- |
| FMDV VP1 N-F | ACCACTTCGACAGGCGAGTCGG |
| FMDV VP1 N-R | CACGTTGGTCAGTGAACCCCCG |
| FMDV VP1 C-F | AGAGGCGATCTCCAAGTGCTGG |
| FMDV VP1 C-R | GATGACCCTGTCTTCCTGGATCAAG |
| FMDV VP1-GFP-F | GTCCGGACTCAGATCTCGAGACCACTTCGACAGGCGAGTCGG |
| FMDV VP1-GFP-R | ATTCGAAGCTTGAGCTCGAGCAAGGACTGCTTTACAGGTGCC |
| swine *Rnf24-*F | TACAGACTAGTACTGGATCCATGAGCTCAGATTTCCCACATT |
| swine *Rnf24*-R | ACTATACGCGTAGTGGATCCTACAATGTTCTCTGCCCCAGGG |
| swine *Lpxn*-F | GTACTGGATCCACTACGCGTATGGAAGAGTTGGATGCCTTGT |
| swine *Lpxn*-R | TCCTTGTCGACTATACGCGTCTGTGTGAAGAGCTTATTGAAG |
| swine *ITGAV*-F | GTACCCGGGCATGCCTCGAGATGGCTTCTCTGCCGCGGCGACGG |
| swine *ITGAV*-R | TTAAGATCTGCTAGCTCGAGTTACCACTTCCTTTGAGTCTTTGA |
| swine *ITGB1*-F | GTACCCGGGCATGCCTCGAGATGAATTTACAACTGATTTTCTGG |
| swine *ITGB1*-R | TTAAGATCTGCTAGCTCGAGTTTTCCCTCATACTTCGGATTGAC |
| *Lpxn* -K157R-F | TGCCAGAGACCGATCGCTGGCAAGATGATCCATGCTCTAGGG |
| *Lpxn* -K157R-R | GCCAGCGATCGGTCTCTGGCAGGAAGCACAATGGCCCTTGGG |
| *Lpxn* -K162R-F | ATCGCTGGCAGAATGATCCATGCTCTAGGGCAAGCGTGGCAT |
| *Lpxn* -K162R-R | ATGGATCATTCTGCCAGCGATCGGCTTCTGGCAGGAAGCACA |
| *Lpxn* -K182R-F | GCTCACTGCAGAGAAGAGATTGGCTCCAGTCCCTTCTTTGAG |
| *Lpxn* -K182R-R | AATCTCTTCTCTGCAGTGAGCGCAGACGAAGTGTTCTGGATG |
| *Lpxn* -K201R-F | GCCTACTGCTCCAGAGACTACCACCACCTTTTTTCTCCACGC |
| *Lpxn* -K201R-R | GTAGTCTCTGGAGCAGTAGGCCAGGCCAGTCCGCTCAAAGAA |
| *Lpxn* -K252R-F | TTTCATGAGAGAGACAAGAAGCCATATTGCCGGAAGGATTTC |
| *Lpxn* -K252R-R | CTTCTTGTCTCTCTCATGAAAACCTTCTGCACCAAACACCTC |
| *Lpxn* -K254R-F | GAGAAGGACAGAAAGCCATATTGCCGGAAGGATTTCTTAGCC |
| *Lpxn* -K254R-R | ATATGGCTTTCTGTCCTTCTCATGAAAACCTTCTGCACCAAA |
| *Lpxn* -K260R-F | TGCCGGAGGGATTTCTTAGCCATGTTTTCACCCAAGTGTGGG |
| *Lpxn* -K260R-R | GGCTAAGAAATCCCTCCGGCAATATGGCTTCTTGTCCTTCTC |
| *Lpxn* *LD*-F | GTACTGGATCCACTACGCGTATGGAAGAGTTGGATGCCTTGT |
| *Lpxn* *LD*-R | TCCTTGTCGACTATACGCGTCTTGGGCACTGTGGCAATTCCG |
| *Lpxn* *LIM*-F | GTACTGGATCCACTACGCGTGGCCATTGTGCTTCCTGCCAGA |
| *Lpxn* *LIM*-R | TCCTTGTCGACTATACGCGTCTGTGTGAAGAGCTTATTGAAG |
| *Lpxn ΔLIM1+2-*F | GGAATTGCCACAGTGCCCAAGCCCAGGTGTGGGGGCTGCAAC |
| *Lpxn ΔLIM1+2-*R | GTTGCAGCCCCCACACCTGGGCTTGGGCACTGTGGCAATTCC |
| *Lpxn ΔLIM3+4-F* | GTACTGGATCCACTACGCGTATGGAAGAGTTGGATGCCTTGT |
| *Lpxn ΔLIM3+4-R* | ACGGGCCCTCTAGACTCGAGTTATGAAAACATGGCTAAGAAA |
| RNF24-KO-F | TAAGGGGCCCCAGGAACTAC |
| RNF24-KO-R | GAAGCTTGTCTGCACTGCCC |

**Table S4 sgRNAs used in this study**

| **sgRNAs ID** | **Sequences (5’-3’)** |
| --- | --- |
| TINAG-sgRNA-F | CACCGTTGAACAAGGCATACGTGCT |
| TINAG-sgRNA-R | AAACAGCACGTATGCCTTGTTCAAC |
| HR-sgRNA-F | CACCGCCGGATACCCCATCACGAGA |
| HR-sgRNA-R | AAACTCTCGTGATGGGGTATCCGGC |
| SGPL1-sgRNA-F | CACCGTCATTGCATGGAGTGTCGTG |
| SGPL1-sgRNA-R | AAACCACGACACTCCATGCAATGAC |
| LCMT2-sgRNA-F | CACCGCGGAACTAGCAAGGCGGCAA |
| LCMT2-sgRNA-R | AAACTTGCCGCCTTGCTAGTTCCGC |
| CDK18-sgRNA-F | CACCGTCCTCGATGGTCTCGGTGCG |
| CDK18-sgRNA-R | AAACCGCACCGAGACCATCGAGGAC |
| REXO5-sgRNA-F | CACCGGCGTCGCGAACCAGCTGGTC |
| REXO5-sgRNA-R | AAACGACCAGCTGGTTCGCGACGCC |
| FNBP1-sgRNA-F | CACCGCGTAGTCATTCATCTCGTTC |
| FNBP1-sgRNA-R | AAACGAACGAGATGAATGACTACGC |
| MEX3B-sgRNA-F | CACCGGCGCGAGCTGTAGGGCTCTT |
| MEX3B-sgRNA-R | AAACAAGAGCCCTACAGCTCGCGCC |
| JUP-sgRNA-F | CACCGATGACTCGGGCATCCACTCG |
| JUP-sgRNA-R | AAACCGAGTGGATGCCCGAGTCATC |
| MAGEB18-sgRNA-F | CACCGAGCACTCCTGAGGCGCCACA |
| MAGEB18-sgRNA-R | AAACTGTGGCGCCTCAGGAGTGCTC |
| UBAP1-sgRNA-F | CACCGGAAGCGGGACTTCAACATGG |
| UBAP1-sgRNA-R | AAACCCATGTTGAAGTCCCGCTTCC |
| ZNF580-sgRNA-F | CACCGCTTGGGGGGCGGTGGGTCCA |
| ZNF580-sgRNA-R | AAACTGGACCCACCGCCCCCCAAGC |
| SFR1-sgRNA-F | CACCGGTAAACCAAGATTTCAAGAT |
| SFR1-sgRNA-R | AAACATCTTGAAATCTTGGTTTACC |
| PCP4-sgRNA-F | CACCGTGGGCAGAAGAAGGTTCAGG |
| PCP4-sgRNA-R | AAACCCTGAACCTTCTTCTGCCCAC |
| UBAP1L-sgRNA-F | CACCGGTATGGATCACAAAGCCTTT |
| UBAP1L-sgRNA-R | AAACAAAGGCTTTGTGATCCATACC |
| GALE-sgRNA-F | CACCGCCGCCGCAAGCTCTCCGGCA |
| GALE-sgRNA-R | AAACTGCCGGAGAGCTTGCGGCGGC |
| ESYT2-sgRNA-F | CACCGGCTTCACGAGGGTCGACGTG |
| ESYT2-sgRNA-R | AAACCACGTCGACCCTCGTGAAGCC |
| BNC2-sgRNA-F | CACCGTGGTTCAGCCGTAGTCGTTC |
| BNC2-sgRNA-R | AAACGAACGACTACGGCTGAACCAC |
| RNF24-sgRNA-F | CACCGCATATCCCCAACTCATCTCG |
| RNF24-sgRNA-R | AAACCGAGATGAGTTGGGGATATGC |
| GALR3-sgRNA-F | CACCGCCAGCAGAGCGCGTAGAGCG |
| GALR3-sgRNA-R | AAACCGCTCTACGCGCTCTGCTGGC |
| TREX1-sgRNA-F | CACCGGGTCACCGTTGTGTGCCACG |
| TREX1-sgRNA-R | AAACCGTGGCACACAACGGTGACCC |
| ATP6V1E2-sgRNA-F | CACCGGGGAGATCGTCGTATATTGA |
| ATP6V1E2-sgRNA-R | AAACTCAATATACGACGATCTCCCC |
| YTHDF2-sgRNA-F | CACCGGCATGAATACTATAGACCAA |
| YTHDF2-sgRNA-R | AAACTTGGTCTATAGTATTCATGCC |

**Table S5 shRNAs used in this study**

| **shRNAs ID** | | **Sequences (5’-3’)** |
| --- | --- | --- |
| LPXN | Sense #1 | CCGGGGAAGGATTTCTTAGCCATGTCTCGAGACATGGCTAAGAAATCCTTCCTTTTT |
|  | Antisense #1 | CCTTCCTAAAGAATCGGTACAGAGCTCTGTACCGATTCTTTAGGAAGGAAAAATTAA |
|  | Sense #2 | CCGGGCTTACTGTGCTGCTCCTATTCTCGAGAATAGGAGCAGCACAGTAAGCTTTTT |
|  | Antisense #2 | CGAATGACACGACGAGGATAAGAGCTCTTATCCTCGTCGTGTCATTCGAAAAATTAA |
|  | Sense #3 | CCGGGCCATATTGCCGGAAGGATTTCTCGAGAAATCCTTCCGGCAATATGGCTTTTT |
|  | Antisense #3 | CGGTATAACGGCCTTCCTAAAGAGCTCTTTAGGAAGGCCGTTATACCGAAAAATTAA |

**Table S6 Primer pairs used for qPCR in this study**

| **Primers ID** | **Sequences (5’-3’)** |
| --- | --- |
| FMDV 3D-F | ACCCGAATGCTGAGTGGATT |
| FMDV 3D-R | TGTTGATGATGCTGGTTGCG |
| *GAPDH*-F | GAGTGAACGGATTTGGCCG |
| *GAPDH*-R | CACCCCATTTGATGTTGGCG |
| *HSPG2*-F | GACATCGCCATGGATACCAC |
| *HSPG2*-R | CAGGACAAGCCAGAATAGCC |
| *ITGAV*-F | TCACAGACTGAAAAGAATGACACA |
| *ITGAV*-R | AGGGTGAGATCCCGCTTAGT |
| *ITGB1*-F | GTCGTGTGTGTGAGTGCAAC |
| *ITGB1*-R | GCTGGGGTAATTTGTCCCGA |
| *ITGB3*-F | ACCAGTAACCTGCGGATTGG |
| *ITGB3*-R | TCCGTGACACACTCTGCTTC |
| *ITGB6*-F | GGGGATTGAACTGCTTTGCC |
| *ITGB6*-R | AGGGCACAGCCACCTTGTA |
| *ITGB8*-F | AAGATTGCTGCTGGTGATGACA |
| *ITGB8*-R | GCTTCTGATAGGCTTCCACTACC |
